# Optical coherence tomography and visual evoked potentials in evaluation of optic chiasm decompression

**DOI:** 10.1038/s41598-022-06097-8

**Published:** 2022-02-08

**Authors:** Pavel Poczos, Tomáš Česák, Naďa Jirásková, Markéta Macháčková, Petr Čelakovský, Jaroslav Adamkov, Filip Gabalec, Jiří Soukup, Jan Kremláček

**Affiliations:** 1 Department of Neurosurgery, University Hospital Hradec Králové, Faculty of Medicine in Hradec Králové, Charles University, Sokolská 581, 500 05 Hradec Králové, Czech Republic; 2 Department of Anatomy, Faculty of Medicine in Hradec Králové, Charles University, Hradec Králové, Czech Republic; 3 Department of Ophthalmology, University Hospital Hradec Králové, Faculty of Medicine in Hradec Králové, Charles University, Hradec Králové, Czech Republic; 4 Department of Otorhinolaryngology and Head and Neck Surgery, University Hospital Hradec Králové, Faculty of Medicine in Hradec Králové, Charles University, Hradec Králové, Czech Republic; 5 4th Department of Internal Medicine-Haematology, University Hospital Hradec Králové, Faculty of Medicine in Hradec Králové, Charles University, Hradec Králové, Czech Republic; 6 The Fingerland Department of Pathology, University Hospital Hradec Králové, Faculty of Medicine in Hradec Králové, Charles University, Hradec Králové, Czech Republic; 7 Department of Medical Biophysics, Faculty of Medicine in Hradec Králové, Charles University, Hradec Králové, Czech Republic; 8 Department of Pathological Physiology, Faculty of Medicine in Hradec Králové, Charles University, Hradec Králové, Czech Republic

**Keywords:** Neuroscience, Medical research, Neurology

## Abstract

Chiasmal compression is a known cause of visual impairment, often leading to surgical decompression of the optic chiasm (OC). A prospective study was held at University Hospital in Hradec Králové to explore sensitivity of optical coherence tomography (OCT) and visual evoked potentials (VEPs) to OC compression and eventual changes after a decompression. 16 patients with OC compression, caused by different sellar pathologies, were included. The main inclusion criterion was the indication for decompressive surgery. Visual acuity (VA), visual field (VF), retinal nerve fibre layer (RNFL) and ganglion cell layer (GCL) thickness, and peak time and amplitude of pattern-reversal (P-VEPs) and motion-onset VEPs (M-VEPs) were measured pre- and postoperatively. The degree of OC compression was determined on preoperative magnetic resonance imaging. For M-VEPs, there was a significant postoperative shortening of the peak time (N160) (p < 0.05). P100 peak time and its amplitude did not change significantly. The M-VEPs N160 amplitude showed a close relationship to the VF improvement. Thinner preoperative RNFL does not present a statistically important limiting factor for better functional outcomes. The morphological status of the sellar region should be taken into consideration when one evaluates the chiasmal syndrome. M-VEPs enable detection of functional changes in the visual pathway better than P-VEPs.

## Introduction

Decompression of the optic chiasm (OC) with subsequent improvement of visual complaints represents one of the main goals of neurosurgical procedures in the treatment of pathologies of the sellar region^1–5^. It is known from practice that surgery is sometimes postponed in asymptomatic incidental small tumoral findings arising in the sellar region and compressing the OC. The “wait-and-see” approach may be chosen especially in such definitive pathologies as pituitary adenomas. A reconsideration of the therapeutic strategy is necessary when visual impairments occur, mainly VF defects^6–9^. But standard automatic perimetry results do not always offer enough information for a surgery indication^10–12^. Hence another tool has been sought to evaluate the functional condition of the visual pathway. Promising data has been reported following the implementation of optical coherence tomography (OCT). In OC compressions, the retinal nerve fiber layer (RNFL) in the peripapillary region is thinner in the temporal and nasal sectors. While some studies point out the importance of measuring the thickness of the RNFL^13,14^, others emphasize the role of gauging of the thickness of the ganglion cell layer (GCL) in the macular region^12,15–19^. Monteiro also found a parallel thickening of the inner nuclear layer (INL)^13^. The Congress of neurological surgeons (2016) gave a recommendation to evaluate the GCL in the macular region to assess a patient’s chances of postoperative vision improvement^21^.

Recent developments in visual evoked potentials (VEPs) including non-standard tests are not yet widespread for the monitoring of visual functions in compression of the OC by tumors. Stimulation of the retina is often achieved by an alternation of black/white checkerboards (pattern-reversal VEPs [P-VEPs]). When the visual stimulation covers symmetrically the left and right visual field (“full field”), the recorded VEPs result from a spatial summation of responses from both brain hemispheres. Stimulation by hemifields is helpful for the monitoring of OC compression. Some works favor the so-called multifocal VEPs (mf-VEPs)^22–25^.

The motion-onset VEPs (M-VEPs) are able to test even more peripheral parts of VF than mf-VEPs (up to 50° excentricity)^26^. This is due to the optimally tuned spatial characteristic of the stimulus for better activation of the magnocellular input of the visual pathway. The receptive fields of the retina for magnocellular inputs are located mainly in the extrafoveal part of the retina.

To the best of our knowledge, no study has been published that would use M-VEPs to evaluate the functional state of the visual pathway in patients with compression of the OC. The main aim of this prospective study is to contribute to the discussion as to whether or not the use of OCT and VEPs has an important role in the planning of treatment and in the preoperative prediction of visual outcomes. Hence, we do offer here an analysis of VEP results and our previously published data subjugated to complementation and revision^27^. Because VF field examination presents the gold standard in visual functions evaluation in a chiasmal syndrome we related the results from OCT and VEPs mainly to VF results.

## Methods

### Patients

All 16 participants (32 eyes) signed an informed consent to procedures conducted in accordance with the Declaration of Helsinki. Participants were recruited from the Department of Neurosurgery and the Department of Ophthalmology of the University Hospital Hradec Králové. The median age of the entire cohort, comprising 8 women and 8 men, was 54 years with an interquartile range (IQR) of 45 to 63 years. Patients underwent four ophthalmological with respective electrophysiological examinations: once preoperatively, and postoperatively at 1 week, 3 months and 6 months. The inclusion criterion was the presence of a tumor in the sellar region and subsequent indication for its surgical removal. The study included patients who underwent surgery from October 2016 to August 2018 at the above-mentioned neurosurgical department. The exclusion criterion was any another disease of the eyes or visual pathway which could have an impact on results obtained by automated perimetry, OCT or VEPs.

### Ophthalmological examination

**Figure 1:**
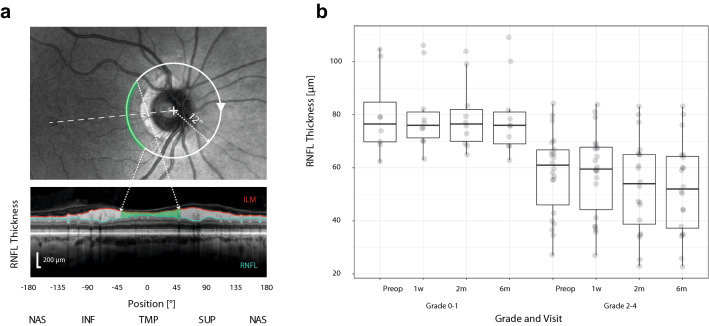
Example from a preoperative OCT examination of patient #8. In the upper panel, the circular scanning trajectory of 3.4 mm diameter centered on the optic nerve disc is depicted on the scanning laser ophthalmoscopy image. The green circular slice represents the temporal part of peripapillary RNFL. In the bottom panel, the green area marks the evaluated temporal RNFL thickness delineated by the internal limiting membrane (ILM) and the interface between the axonal fibers and the bodies of the ganglion cells (**a**). Distribution of the temporal RNFL thickness in preoperative and three postoperative visits separately for patients with a grade of compression 0–1 and 2–4. The grade factor was statistically significant (p = 0.180 × 10^–6^). The lower and upper hinges of the boxplots represent the 25th and 75th percentiles, respectively; the whiskers extend to an outlier but not farther than 1.5 times the interquartile range. The boxplot overlays the individual values represented as gray circles spread along the horizontal dimension to avoid overlapping (**b**).

**Figure 2:**
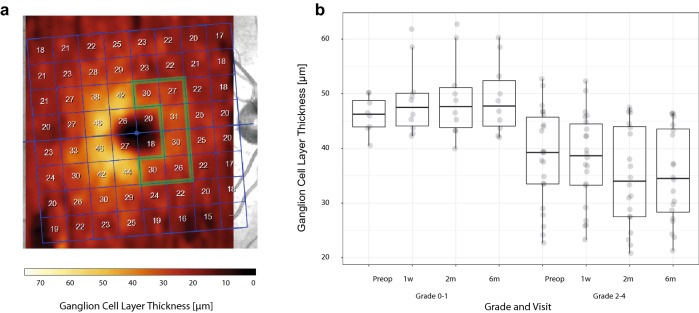
Example from a preoperative OCT examination of patient #8. The limited field (inside the green line) represents the nasal half of the fovea used for the quantification of the ganglion cell layer (GCL) thickness. The GCL thickness is color-coded and clearly shows hemifield asymmetry related to the chiasmatic syndrome (**a**). The distribution of GCL thickness was significantly different between patients with the grade of compression 0–1 and 2–4 (p = 0.024 × 10^–6^). For the description of the boxplot parameters, see the legend of Fig. 1b (**b**).

Landolt circles were used for the examination of distant visual acuity (VA). The visual field was examined using a Humphrey Field Analyzer II (Carl Zeiss, Meditec, Dublin, CA, USA; Test: SITA Strategy—FAT, Central 30-2 Threshold Test). For the purposes of statistical evaluation, the mean deviation (MD), giving an overall value of the total amount of VF loss, was monitored. The measurements of thickness of RNFL and GCL were performed on a Spectralis OCT system (Heidelberg Engineering, Heidelberg, Germany; Heidelberg Eye Explorer 1.10.2 software). Given the very purpose of the study, retinal areas related to crossing fibers of the OC were mainly evaluated. The thickness of RNFL centered on the optic nerve disc was measured along a 3.4 mm diameter circle and the temporal segment (± 45°) was selected for subsequent data processing (Fig. 1a). The ganglion cell layer (GCL) thickness was quantified from the nasal half of a 3.45 × 4.15 mm rectangle centered on the fovea. The central area (1.73 × 2.08 mm) was excluded from evaluation (Fig. 2a).

### Electrophysiological examination

**Figure 3:**
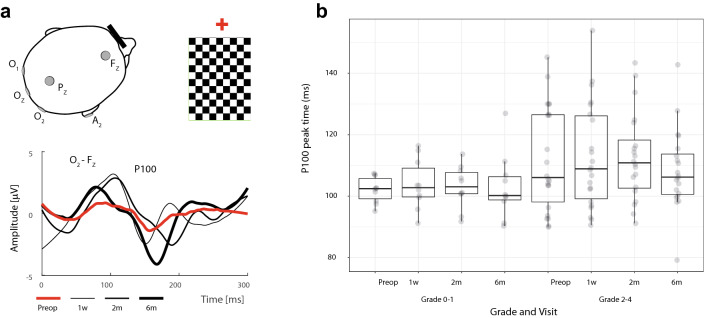
Schema illustrating the recording of pattern-reversal visual evoked potentials (P-VEPs) from the right eye fixating the red cross (left from the checkerboard) in patient #8. Such was the way the crossed fibers were examined. Active electrodes (O_1_, O_z_ and O_2_) and the reference electrode (F_z_) were placed according to the 10–20 electroencephalographic system. The different waveforms represent preoperative (red line) and different postoperative (at 1 week, 3 months and 6 months) P-VEPs in O_2_–F_Z_ derivation (the paradoxical lateralization taken into consideration) (**a**). Distribution of the P100 amplitude in P-VEPs between patients with the grade of compression 0–1 and 2–4. The grade was not a significant factor (p = 0.195). For the description of the boxplot parameters, see the legend of Fig. 1b (**b**).

**Figure 4:**
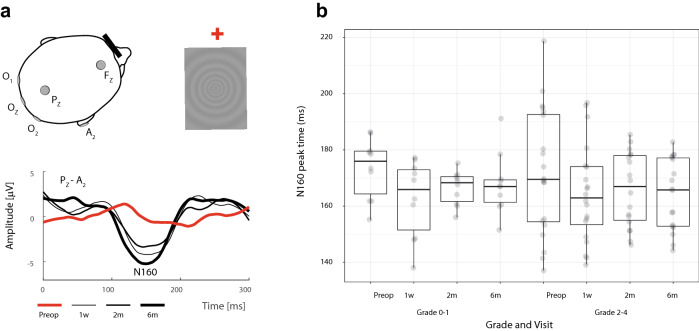
Schema illustrating the recording of motion-onset visual evoked potentials (M-VEPs) from the right eye fixating the red cross (left from the pattern) in patient #8. Such was the way the crossed fibers were examined. Active electrodes (O_1_, O_z_, O_2_ and P_z_) and the reference electrode (A_2_) were placed according to the 10–20 electroencephalographic system. The different wave forms represent preoperative (red line) and different postoperative (at 1 week, 3 months and 6 months) M-VEPs in P_Z_–A_2_ (**a**). Distribution of the N160 amplitude in M-VEPs between patients with grade of compression 0–1 and 2–4. The grade was not a significant factor (p = 0.696). For the description of the boxplot parameters, see the legend of Fig. 1b (**b**).

Visual evoked potentials were recorded on a Synergy Medelec device (VIASYS Healthcare, Inc., USA). Pattern-reversal and motion-onset stimuli were used for stimulation of photoreceptor cells. In each eye, the nasal and temporal half of the retina were repeatedly stimulated (2 × 100 stimulations), separately for P-VEPs and M-VEPs. To evoke the P-VEPs and M-VEPs, the stimuli consisted respectively of alternation of black and white squares of checkerboards (Fig. 3a), and of gray concentric circles that expanded and contracted (Fig. 4a).

The stimuli parameters for P-VEPs were 2 Hz reversal frequency, 85% contrast, average luminance 40 cd/m^2^, lateral hemifield stimulation (11° × 14°), and check element size of 60'. Different parameters were used for M-VEPs: low contrast of 14%, average luminance 40 cd/m^2^, temporal frequency constant over the whole area of 5 cycles per second, stimulus duration 100 ms, and interstimulation interval 400 ms. The stimulation area was the same as for P-VEPs.

The record of monocular P-VEPs was obtained from three channels relative to a reference electrode placed in the Fz position according to the 10–20 electroencephalographic system^28^. Active electrodes were then placed in positions O1, Oz and O2. Derivations in positions O1, O2, Oz and Pz were used for monocular M-VEPs. The unipolar record had a reference electrode on the right earlobe (Fig. 3a and 4a). The grounding electrode was always on the right wrist. Due to paradoxical lateralization, the P-VEPs were evaluated from the ipsilateral electrode (Fig. 3a)^29^. Motion VEPs were assessed from the Pz electrode^30^ because their lateralization is not uniform with respect to the stimulated part of the VF^26^, and in adults is asymmetric irrespective of hemifield stimulated (Fig. 4a)^31^. Given the very purpose of the study, results obtained by stimulating the nasal halves of the retina were evaluated.

VEPs filtering, extraction of the parameters of interest, and data plotting were conducted in MATLAB Release 2019b (MathWorks, USA). For the VEP assessments, authors (PP and JK) overlapped single-patient VEPs from all visits and marked appropriate peaks for the group of curves (Fig. 3a and 4a). Subsequently, peaks for a particular visit were automatically identified as a local extreme within an interval ± 25 ms from the location the markers were placed. This semiautomatic process ensured that the authors were blind to the visit order and their bias was minimized.

### Analysis of preoperative MR

The degree of OC compression, the so-called grade, was determined on coronal sequences of T1-weighted images of preoperative MR (magnetic resonance imaging) based on criteria proposed by Fujimoto^32^. In grade 0 there was no contact between the tumor and the OC. Minimal contact without upper OC surface deformity characterized grade 1. Grade 2 indicated contact with upper surface deformity of OC and visible chiasmatic cistern. Grade 3 demonstrated the same as grade 2, but the cistern was invisible, but nevertheless with no cerebral deformity. In the case of grade 4 the contact was so severe that cerebral deformity was clearly noted.

### Surgery

The same operating team performed all decompressive procedures. Transcranial surgery was elected in two (12.5%) patients. In the remaining 14 surgeries, decompression was achieved via the transnasal transsphenoidal route, in which a microscope was used in five of the cases and an endoscope in nine.

### Statistical analysis

Statistical analysis was performed using the software R 3.5.1^33^. Using the Anderson–Darling test (“nortest” package), we tested the normality of the data distribution. Data evaluation and interpretation is related to eyes and not to patients, mainly because the lesions do not symmetrically compress the OC or the respective optic nerves. Another reason is that crossing fibers are compressed mostly just anterior to the OC^34^. Analysis of results from individual eyes rather from the combined results from a patient helps to achieve statistically more robust conclusions. We present descriptive results as the median and interquartile range. To compare values pre- and post-surgery, we used the Wilcoxon signed rank test or paired *t* test for data with respectively a non-normal or a normal distribution. Relationships between continuous markers of interest were calculated using Pearson’s correlation coefficient or Spearman’s rank correlation, depending on the normality of the data distribution. The level of statistical significance was preset to p < 0.05.

For post-hoc analysis of the results, patients were divided into two groups, either with no or minimal OC pressure (grades 0–1) or with unambiguous OC pressure (grades 2–4), and a variability analysis was performed. Kruskal–Wallis nonparametric test was used to assess differences among the groups and visits in cases where data were found to have non-normal distribution by Anderson–Darling test or inhomogeneity of variances by Bartlett test, while ANOVA was used for normally-distributed data with homogeneous variances.

### Ethics approval

This project was approved by the Ethics Committee of the University Hospital Hradec Králové (reference number 201702 S14P).

## Results

### Tumor types

Pathological analysis revealed a non-functioning pituitary adenoma in 5 cases (31.25%), meningioma in two cases (12.5%), both prolactin and growth hormone-producing adenoma twice (12.5%), and a single case each of somatotropic adenoma (6.25%), corticotropic adenoma (6.25%), granular cell tumor of the infundibulum (6.25%), and spindle-cell oncocytoma (6.25%). A single case each of post-infectious cyst (6.25%), tension arachnoid cyst (6.25%), and pituitary apoplexy (6.25%) were also present.

### Visual acuity and visual field

Preoperative and postoperative medians and IQR of VA (logMAR) and VF (mean deviation—MD) are shown in Table 1. The results clearly indicate a tendency of continuous improvement in VA and VF in the postoperative period. To note that 8 patients did not have subjective visual impairment preoperatively.

**Table 1:** Summary of the medians and interquartile ranges of measured parameters (VA, VF, OCT, VEPs) preoperatively and postoperatively.

	Preop	1w	3m	6m
VA [logMAR]	0.4 [0.1; 0.72] n = 32	0.3 [0.1; 0.63]^#^ n = 32	0.3 [0.0; 0.4]^###^ n = 30	0.24 [0.1; 0.25]*** n = 28
VF [MD]	− 4.82 [− 14.25; − 1.84] n = 28	− 2.68 [− 6.06; 0,79]^###^ n = 28	− 0.99 [− 3.4; − 0.37]^###^ n = 30	− 1.17 [− 2.93; − 2.07]^###^ n = 30
RNFL [µm]	65.0 [56.25; 73.0] n = 30	65.5 [56.75; 73.75] n = 30	65.0 [46.75; 74]** n = 28	61.5 [44; 73] *** n = 28
GCL [µm]	43.45 [35.47; 46.65] n = 30	42.3 [35.98; 45.95] n = 30	41.35 [30.45; 46.5]^##^ n = 28	42.15 [30.18; 45.92]^#^ n = 28
P100 L [ms]	104.55 [98.18; 117.98] n = 32	105.6 [99.22; 115.72] n = 32	107.7 [100.88; 114.15] n = 30	105.15 [99.9; 111.52] n = 30
P100 A [µV]	1.94 [1.24; 3.04] n = 32	2.12 [1.76; 3.06] n = 32	2.46 [1.77; 3.28] n = 30	2.3 [1.75; 3.03] n = 30
N160 L [ms]	172.8 [156.98; 184.57] n = 30	163.35 [150.52; 173.85]* n = 30	168.3 [156.75; 175.35]** n = 28	166.35 [156.68; 176.85]* n = 28
N160 A [µs]	4.01 [2.7; 5.05] n = 30	4.42 [3.77; 5.22] n = 30	4.26 [2.93; 5.11]* n = 28	3.91 [2.98; 5.12] n = 28

*VA* visual acuity, *VF* visual field, *GCL* ganglion cell layer, *RNFL* retinal nerve fiber layer, *L* latency (peak time), *A* amplitude, *Preop* preoperatively, *w* week (postoperatively), *m* month (postoperatively), ^#^p < 0.05, ^##^p < 0.01, ^###^p < 0.001 (Wilcoxon signed-rank test), *p < 0.05, **p < 0.01, ***p < 0.001 (paired *t* test). For more detailed statistical analysis see Supplementary Information.

### OCT

The median preoperative and postoperative thicknesses of temporal RNFL and nasal GCL in all eyes and their IQR are listed in Table 1. At the second and third postoperative examination the temporal RNFL and likewise the nasal GCL clearly became significantly thinner. Figures 1a and 2a show the areas of analysis for the temporal RNFL and nasal GCL (in patient #8).

### VEP

Data are related to the crossed fibers. Table 1 offers the median and IQR of preoperative and postoperative peak times (also known as implicit peak time or latency) and amplitudes for P100 and N160. P100 peak time tended to be shorter only at the third follow-up when comparing with preoperative data (p = 0.179, Wilcoxon signed-rank test). All postoperative examinations showed a higher amplitude of P100 without statistical significance. In the case of M-VEPs (N160), there was a statistically significant shortening of the peak time at all postoperative follow-ups and increasing of the amplitude at the second follow up.

### Bitemporal hemianopia

Eight patients (50%) presented bitemporal hemianopia. Visual acuity and VF were significantly better in cases without bitemporal hemianopia (p = 0.001 × 10^–3^ and p = 0.034 × 10^–3^, Wilcoxon rank sum exact test). Temporal RNFL and nasal GCL thickness were statistically significantly lower in patients with bitemporal hemianopia (p = 0.180 × 10^–6^ and p = 0.002 × 10^–3^) in all 4 examinations. Also, there were more eyes with thinner GCL in the group of patients with bitemporal hemianopia preoperatively (p = 0.679 × 10^–3^). No statistically significant difference was found when comparing patients with and without bitemporal hemianopia in any of the P-VEP and M-VEP parameters (no intergroup difference using Kruskal–Wallis nonparametric ANOVA, p > 0.195).

### Grade

In two patients there was no visible OC compression on preoperative imaging (12.5%). Grades 1, 2, 3 and 4 were present in three (18.75%), three (18.75%), two (12.5%), and six (37.5%) cases, respectively.

The preoperative median of VA was significantly better in the group of patients with compression grade 0–1 (logMAR 0.048) in comparison to patients with grade 2–4 (logMAR 0.602). Also, the extent of preoperative VF was better in group 0–1 (median of MD − 1.535) than in group 2–4 (− 8.885).

Patients with no or minimal compression (grade 0–1) presented a preoperatively higher median of temporal RNFL (76.5 µm) and nasal GCL (46.3 µm) than the cohort with grade 2–4 (61.0 µm and 39.3 µm) (p = 0.180 × 10^–6^ and p = 0.024 × 10^–6^, paired *t* test) (Fig. 1b and 2b). Measurements did not reveal any statistically important preoperative differences in peak time for P-VEPs and M-VEPs when comparing the groups grade 0–1 (102 and 176 ms, respectively) and grade 2–4 (106 (p = 0.210) and 167 ms (p = 0.579), respectively). No intergroup difference was observed when amplitudes of P-VEPs and M-VEPs were compared (2.9 µV and 4.1 µV versus 1.7 µV (p = 0.195) and 3.9 µV (p = 0.696)) (Figs. 3b, 4b).

### Correlation analysis

**Figure 5:**
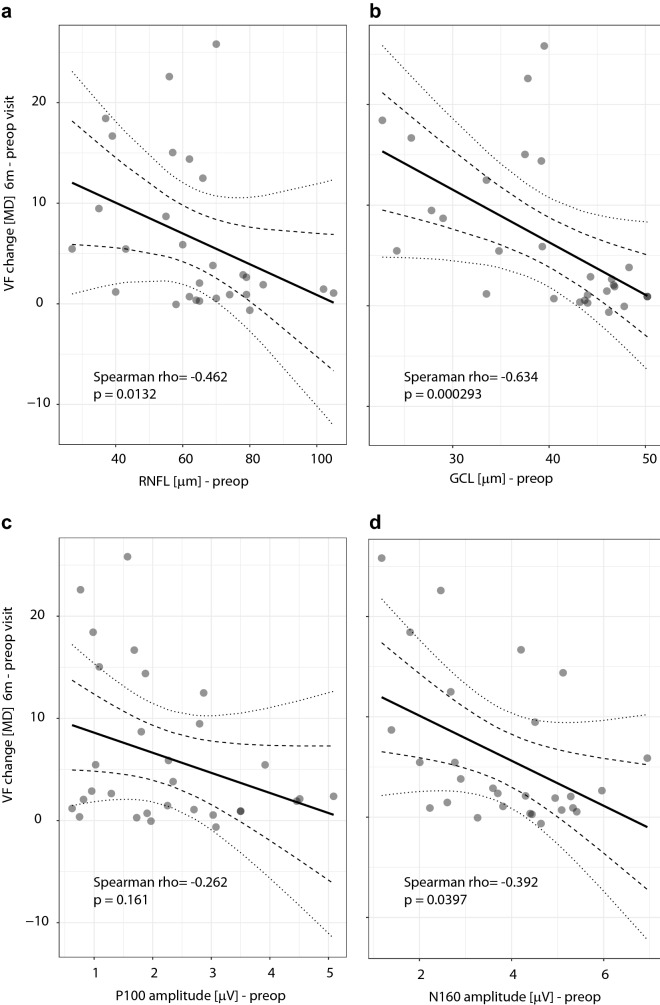
Plots illustrating the relationship between VF improvement and selected OCT (**a**,**b**) and VEPs (**c**,**d**) parameters. Higher is better for VF improvement, which was calculated as the difference between the last and the preoperative value of mean deviation (MD). The single dot represents one eye; the thick linear regression line is surrounded by dashed 99.9% and dotted 95.0% confidence intervals. A negative dependency between the VF improvement and preoperative state is evident in all plots. Such relationship illustrates a situation where the more affected initial state of the visual system is related to a higher benefit from the treatment. To note a statistically significant relationship between the improvement of and N160 amplitudes.

The correlation analysis illustrates a clear negative dependency between preoperative thickness of the temporal RNFL and improvement of VA and VF (Fig. 5a) at the time of the third follow-up (Spearman rho = 0.542; p = 0.004 and Spearman rho = − 0.462; p = 0.013). Similar dependency was noted for GCL with p = 0.008 (Spearman rho = 0.505) and p = 0.293 × 10^–3^ (Spearman rho = − 0.639) (Fig. 5b).

Correlation analysis indicates a relationship between preoperative P-VEPs (Fig. 5c)/M-VEPs parameters and the VA and VF improvement calculated as the difference between the last and the preoperative value. There was a statistically significant relationship only between the improvement of MD and N160 amplitudes (Spearman rho = − 0.392; p = 0.038) (Fig. 5d).

## Discussion

Authors have attributed the preoperative extent of the VF deficit as the major influence on its recovery^13–35^. However, the VF examination is burdened by an element of subjectivity and has not always been sufficiently sensitive to identify VF defects^10–12^. This led us to the idea of evaluating the potential of OCT and VEPs, mainly the M-VEPs, in the management of OC compression.

Earlier studies have affirmed that preoperative average RNFL thickness below 70–85 µm is a negative prognostic factor for both immediate and long-term visual improvement^36–38^. However, Loo et al. admitted that visual recovery may occur even with average peripapillary RNFL of less than 70 µm^38^. Based on the negative correlation between preoperative thickness of RNFL, either temporal or average, and improvement in VA/VF (Fig. 5a), our results tend to show that lower average preoperative RNFL thickness (< 85 µm; to note the absence of a clear-cut) is related to a greater postoperative visual improvement. In general, the observed dependencies were more obvious when the temporal RNFL was taken into consideration. It reflects better the effort to evaluate the crossing fibers of the OC. The preoperative temporal RNFL thicknesses of our patients were scattered around the lower values (median 65 µm), and we did not observe an RNFL threshold limiting the functional improvement.

More recent studies have accentuated the role of the GCL, or GCC (Ganglion Cell Layer Complex). Some works have highlighted that binasal thinning of the GCC often corresponds with a bitemporal depression in the VF^12,16–18,20,39,40^. Paradoxically, Yoneoka presented a result of a stronger correlation with the RNFL^41^. An analysis of our results shows a more significant concordance between preoperative nasal GCL and VF defects (Spearman rho = 0.678; p = 0.039 × 10^–5^) in comparison to temporal RNFL (Spearman rho = 0.559; p = 0.001). This could be explained by less precise relationship between deficits on standard automated perimetry and RNFL loss, than in the GCL loss. The negative correlation between preoperative nasal GCL and VF improvement (Fig. 5b) is stronger in contrast with the temporal RNFL. Nasal GCL thickness less than 40 µm (without a clear-cut) tends to have a bigger functional benefit for postoperative visual functions. Given the complex structure underlying visual perception, RNFL or GCL thickness might not fully correspond to the visual impairment. For a more accurate assessment of the impact of the preoperative RNFL or GCL thickness on postoperative visual changes, a group of patients with similar visual deficit should be examined.

A progressive thinning of RNFL and GCL, with interindividual differences, is documented by our data despite a significant improvement in the VF. An ongoing process of nerve fiber degeneration serves as an explanation. Neither should be excluded manipulation of the optic nerves (during tumor resection) as a plausible exogenic determinant for axonal degeneration.

The use of VEPs to monitor visual functions in the case of optic nerve or OC compressions has not been as widespread as for optic neuritis or glaucoma^22,24,25,42–45^. The low sensitivity of standard VEPs to OC compression/decompression was enhanced by sequential stimulation of smaller parts of the visual field (mf-VEP) at the cost of prolonging the examination, which increased the demands on the patient’s attention. Two teams showed a statistically significant and strong correlation between depressions in the temporal half of the VF and mf-VEP parameters^22,25^.

Conventional VEPs (e.g., P-VEPs) are able to harvest responses from approximately the central 15° of the VF^46^. This means that those protocols are not able accurately to display the spatial details and peripheral affections of the perimeter. A more objective evaluation of the functional integrity of the visual pathway is achieved with mf-VEPs that encompass 25°–32° of the VF^47,48^. M-VEPs, under certain stimulation conditions, offer the possibility for testing even more peripheral parts of the VF (up to 50° eccentricity)^26^. Using low spatial frequency stimulation in the periphery of the VF activates predominantly magnocellular visual input, which helps to achieve such a wide range. Activation of this part of the visual pathway may provide a different sensitivity than the P-VEPs. The receptive fields of the retina for magnocellular input information (parasol ganglion cells) are found mainly in the extrafoveal part of the retina. This suggests that the M-VEPs might be more helpful and accurate in testing patients with minimal peripheral VF abnormalities. The same applies for mf-VEPs. It has not been possible to compare our M-VEPs results with those from other works. To the best of our knowledge, no publication has yet described the use of M-VEPs to assess the functional state of the visual pathway in OC compression. In all three postoperative examinations, in most cases M-VEPs showed statistically significant shortening of peak time and increasing of amplitude (Table 1). Such evident results were not obtained in P-VEPs, supporting the fact that M-VEPs have a higher sensitivity to stimulation in the peripheral areas of the retina.

Correlation analysis has demonstrated only few relationships between preoperative and postoperative parameters of P-VEPs/M-VEPs and VA or MD improvement (Fig. 5c,d). A statistically significant relationship was observed only between improvement of MD and N160 amplitudes (Spearman rho = − 0.392; p = 0.038) (Fig. 5d). No statistically significant differences were found in any of the P-VEPs and M-VEPs parameters when comparing patients with or without bitemporal hemianopia. Four of those 8 patients without preoperative subjective visual impairment had a normal VF examination. In one case, M-VEPs reflected an obvious pathological finding of the crossed fibers of the right eye (patient #8). Preoperatively, an increase in the N160 peak time was apparent on the right eye. This was subsequently improved in the postoperative period.

Results from a perimeter or OCT do not present unequivocal prognostic parameters. The little-discussed preoperative variable, the degree of OC compression, has a strong impact on the indication for surgery and the postoperative visual outcome^49^. Our results show that the mean thickness of the RNFL (Fig. 1b) as well as of the GCL (Fig. 2b) was statistically significantly greater in grade 0–1 than in grade 2–4. However, it must be emphasized that there were patients with a significant OC compression but who had a satisfactory thickness of RNFL and GCL. This underlines the concept of multifactorial cause of chiasmal syndrome (direct forces acting on OC and/or disturbed blood supply leading to local ischemia at the level of the OC)^11,50^.

When comparing patients with grade of compression 0–1 with those with grade 2–4, the median preoperative latencies were not statistically significantly different for P-VEPs and M-VEPs. In other words, there was no peak time prolongation in patients with greater OC compression. In both groups, there was a shortening of N160 peak time in the first postoperative control. However, for other follow-up controls, the peak time values were close to preoperative values. We attribute the improvement at the first postoperative control mainly to the effect of decompression. Conversely, subsequent progressive mild “deterioration” of the parameters could be explained by slow postoperative changing of traction forces (as a component of scarring) acting on the OC, or by continued antero- and retrograde degeneration^51,52^. Although less probably, it could be attributed to the manipulation of the optic nerves (during tumor resection). In summary there were no important preoperative pathological VEP changes in the case of the higher grades. Again, it indicates that the etiology of chiasmal syndrome has more components than solely morphology.

Our unique findings with radial motion stimulation in patients with OC compression suggest that M-VEPs are able to detect functional changes resulting from compression of those optic nerve fibers that carry information from the peripheral parts of the retina. Radial movement tends to be the most effective way of stimulation^53^. This is mainly because the stimulus design respects the cortical magnification factor, and probably also because this type of motion resembles the optic flow, which is present during observer self-motion through an environment.

The study has several limitations. Assuming the greatest impact of a tumor compression is on crossed fibers, as seen in the nasal GCL, we chose the temporal RNFL segment for preferential evaluation. However, the average (global) RNFL appeared to have a strongest link to changes in VF and VA. As already mentioned, probably it is due to the poorer retinotopic mapping of the RNFL. VEP examination generally has a high sensitivity to factors influencing the test results (pupil diameter, refractive error, age, sex, electrode position, anatomical variations, cortical excitability, etc.). So we expected a high VEP response to the OC compression/decompression; however, we did not observe this in our sample. This decreased sensitivity is likely due to the suboptimal stimulation, where stimulation patterns were projected only in a small field (11° × 14°) laterally from the center of the fovea. This weakens the outcome, mainly of P-VEPs. The VEPs examination, which requires systematic attentive cooperation from patients, may contribute to possible bias of the results, because patients may become tired during the examination. While all patients were cooperative in our group, in less cooperative patients, mf-VEPs were considered more reliable than the VF examination^54^. The group of 32 eyes represents a relatively small cohort. In addition, some patients lacked a complete series of all three follow-up postoperative controls.

## Conclusions

Our results and the available literature sources show that VF, OCT and the respective VEP examinations should be viewed as complementary methods for providing essential information about the morphological and functional state of the visual pathway, rather than as competitive. VEPs reveal visual impairment in patients without subjective complaints and with minimal OC compression. This is the first prospective study of the use of M-VEPs in addition to P-VEPs to detect functional changes in the visual pathway in the follow-up of patients with OC compression. The presented results favour morphological data about the retinal status (from OCT) as having greater potential for predicting postoperative development than an alteration of the visual function measured by VEPs.

## Supplementary Information

Supplementary Information.

## Data Availability

All data collected are available upon request to the corresponding author.
