# Supplementary material for: Optical coherence tomography and visual evoked potentials in evaluation of optic chiasm decompression
Source: Sci Rep. 2022 Feb 8;12:2102. doi: 10.1038/s41598-022-06097-8 (PMC8825827; doi:10.1038/s41598-022-06097-8)
Supplement: Supplementary file 1 — Supplementary Information. [file 41598_2022_6097_MOESM1_ESM.docx]

Optical coherence tomography and visual evoked potentials in evaluation of optic chiasm decompression

Pavel Poczos, Tomáš Česák, Naďa Jirásková, Markéta Macháčková, Petr Čelakovský, Jaroslav Adamkov, Filip Gabalec, Jiří Soukup, Jan Kremláček

December, 14^th^, 2021

Table of Contents

Descriptive stat. 3

Age 3

Grade 3

Pre- vs post- treatment 4

RNFL before surgery 1 visit after surgery 4

RNFL before surgery 2 visit after surgery 6

RNFL before surgery 3 visit after surgery 8

GCL before surgery 1 visit after surgery 10

GCL before surgery 2 visit after surgery 11

GCL before surgery 3 visit after surgery 12

P100 peak time before surgery 1 visit after surgery 14

P100 peak time before surgery 2 visit after surgery 16

P100 peak time before surgery 3 visit after surgery 18

P100 amplitude before surgery 1 visit after surgery 20

P100 amplitude before surgery 2 visit after surgery 22

P100 amplitude before surgery 3 visit after surgery 24

N160 peak time before surgery 1 visit after surgery 26

N160 peak time before surgery 2 visit after surgery 28

N160 peak time before surgery 3 visit after surgery 30

N160 amplitude before surgery 1 visit after surgery 32

N160 amplitude before surgery 2 visit after surgery 34

N160 amplitude before surgery 3 visit after surgery 36

Visual acuity before surgery 1 visit after surgery 38

Visual acuity before surgery 2 visit after surgery 40

Visual acuity before surgery 3 visit after surgery 42

Perimetry before surgery 1 visit after surgery 44

Perimetry before surgery 2 visit after surgery 46

Perimetry before surgery 3 visit after surgery 48

Grade 50

RNFL 50

GCL 52

P100 peak time 54

P100 amplitude 56

N160 peak time 58

N160 amplitude 60

Visual acuity 62

Perimetry 64

Visual field change 66

RNFL 66

GCL 68

P100 peak time 70

P100 amplitude 72

N160 peak time 74

N160 amplitude 76

Visual acuity 78

# Descriptive statistics

##

## Age

**Table 1**Descriptive statistics for the age of patients. Parameters listed: N – number of observations, 25q – Lower quartile, Median, 75q – Upper quartile, Mean, SD – Standard deviation, Min – Minimal value, Max – Maximal value, NA – not available.

|  | N | 25q | Median | 75q | Mean | SD | Min | Max | NA |
| --- | --- | --- | --- | --- | --- | --- | --- | --- | --- |
| Age | 32 | 44.94 | 53.615 | 62.67 | 51.939 | 11.86 | 23 | 66 | 96 |

##

## Grade

***Table 2***Number of patients (Freq) with given Grade.

| Grade | Freq |
| --- | --- |
| 0 | 4 |
| 1 | 6 |
| 2 | 6 |
| 3 | 4 |
| 4 | 12 |

#

# Pre- vs post- treatment

Paired comparisons. Values for VA = 0 were replaced by 0.01. The corresponding logMAR = 2 / by WHO it corresponds to blindness.

### RNFL before surgery 1 visit after surgery

*Two tail tests* Anderson-Darling test: normality = TRUE

**NO** paired difference, ttest p= 0.8914139

Effect size and confidence limits, d = 0.04 [ -0.47 0.54 ]

Power of study with aforementioned effect, pwr = 0.05

RNFL before surgery (um) vs. RNFL 1 visit after surgery(um)

**Table 3**Descriptive statistics for the RNFL before and after surgery. Parameters listed*: N*– *number of observations, 25q – Lower quartile, Median, 75q* – *Upper quartile, Mean, SD – Standard deviation, Min – Minimal value, Max – Maximal value, NA – not available.*

|  | N | 25q | Median | 75q | Mean | SD | Min | Max | NA |
| --- | --- | --- | --- | --- | --- | --- | --- | --- | --- |
| RNFL before surgery (μm) | 30 | 56.25 | 65.0 | 73.00 | 63.93 | 18.11 | 27 | 105 | 0 |
| RNFL 1 visit after surgery(μm) | 30 | 56.75 | 65.5 | 73.75 | 63.90 | 18.44 | 27 | 106 | 0 |
| Diff. | 30 | -1.00 | 0.0 | 1.00 | 0.03 | 1.33 | -2 | 3 | 0 |


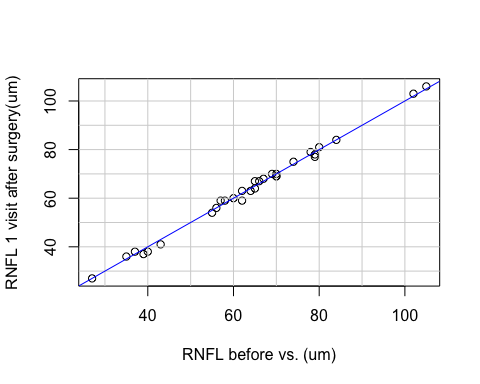


**Figure 1**Scatter plot for RNFL before vs. the first visit after surgery. Each dot represents one eye, the blue link depicts the identity line.

### RNFL before surgery 2 visit after surgery

*Two tail tests* Anderson-Darling test: normality = TRUE

**SIGNIFICANT** paired difference, ttest p= 0.003574899

Effect size and confidence limits, d = 0.81 [ 0.27 1.36 ]

Power of study with aforementioned effect, pwr = 0.86

RNFL before surgery (um) vs. RNFL 2 visit after surgery(um)

**Table 4**Descriptive statistics for the RNFL before and after surgery. Parameters listed: *N*– *number of observations, 25q – Lower quartile, Median, 75q* – *Upper quartile, Mean, SD – Standard deviation, Min – Minimal value, Max – Maximal value, NA – not available.*

|  | N | 25q | Median | 75q | Mean | SD | Min | Max | NA |
| --- | --- | --- | --- | --- | --- | --- | --- | --- | --- |
| RNFL before surgery (um) | 28 | 55.75 | 64.5 | 75 | 63.61 | 18.72 | 27 | 105 | 0 |
| RNFL 2 visit after surgery(um) | 28 | 46.75 | 65.0 | 74 | 60.61 | 20.46 | 23 | 104 | 0 |
| Diff. | 28 | 0.00 | 1.0 | 4 | 3.00 | 4.97 | -3 | 17 | 0 |


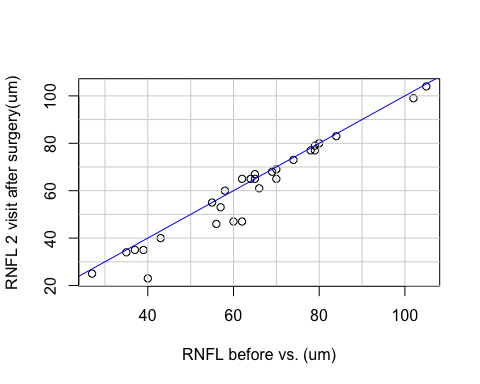


**Figure 2**Scatter plot for RNFL before vs. the second visit after surgery. Each dot represents one eye, the blue link depicts the identity line.

### RNFL before surgery 3 visit after surgery

*Two tail tests* Anderson-Darling test: normality = TRUE

**SIGNIFICANT** paired difference, ttest p= 0.000553404

Effect size and confidence limits, d = 0.98 [ 0.43 1.54 ]

Power of study with aforementioned effect, pwr = 0.96

RNFL before surgery (um) vs. RNFL 3 visit after surgery(um)

**Table 5**Descriptive statistics for the RNFL before and after surgery. Parameters listed: *N– number of observations, 25q – Lower quartile, Median, 75q – Upper quartile, Mean, SD – Standard deviation, Min – Minimal value, Max – Maximal value, NA – not available.*

|  | N | 25q | Median | 75q | Mean | SD | Min | Max | NA |
| --- | --- | --- | --- | --- | --- | --- | --- | --- | --- |
| RNFL before surgery (um) | 28 | 55.75 | 64.5 | 75.00 | 63.61 | 18.72 | 27 | 105 | 0 |
| RNFL 3 visit after surgery(um) | 28 | 44.00 | 61.5 | 73.00 | 59.96 | 20.90 | 23 | 109 | 0 |
| Diff. | 28 | 1.00 | 2.0 | 5.25 | 3.64 | 4.92 | -4 | 16 | 0 |


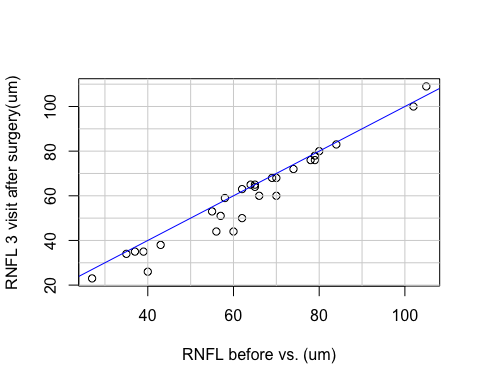


**Figure 3**Scatter plot for RNFL before vs. the third visit after surgery. Each dot represents one eye, the blue link depicts the identity line.

### GCL before surgery 1 visit after surgery

*Two tail tests* Anderson-Darling test: normality = TRUE

**NO** paired difference, ttest p= 0.1840582

Effect size and confidence limits, d = 0.35 [ -0.16 0.86 ]

Power of study with aforementioned effect, pwr = 0.27

GCL before surgery (um) vs. GCL 1 visit after surgery(um)

**Table 6**Descriptive statistics for the GCL before and after surgery. Parameters listed: *N – number of observations, 25q – Lower quartile, Median, 75q – Upper quartile, Mean, SD – Standard deviation, Min – Minimal value, Max – Maximal value, NA – not available.*

|  | N | 25q | Median | 75q | Mean | SD | Min | Max | NA |
| --- | --- | --- | --- | --- | --- | --- | --- | --- | --- |
| GCL before surgery (um) | 30 | 35.47 | 43.45 | 46.65 | 40.57 | 8.41 | 22.7 | 52.7 | 0 |
| GCL 1 visit after surgery(um) | 30 | 35.98 | 42.30 | 45.95 | 40.24 | 8.06 | 23.3 | 52.3 | 0 |
| Diff. | 30 | -0.28 | 0.35 | 1.00 | 0.33 | 1.33 | -3.5 | 4.0 | 0 |


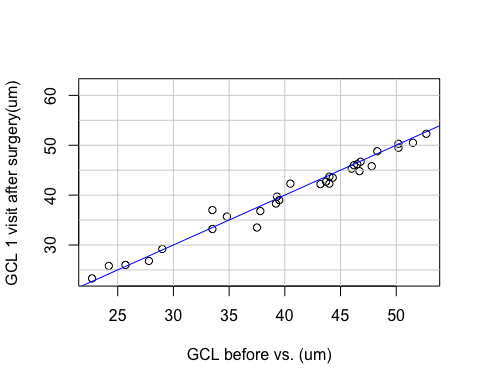


**Figure 4**Scatter plot for GCL before vs. the first visit after surgery. Each dot represents one eye, the blue link depicts the identity line.

### GCL before surgery 2 visit after surgery

*Two tail tests* Anderson-Darling test: normality = FALSE

**SIGNIFICANT** paired difference, Wilcoxon p= 0.002354281

Effect size and confidence limits, d = 0.85 [ 0.31 1.4 ]

Power of study with aforementioned effect, pwr = 0.89

GCL before surgery (um) vs. GCL 2 visit after surgery(um)

**Table 7**Descriptive statistics for the GCL before and after surgery. Parameters listed: *N– number of observations, 25q – Lower quartile, Median, 75q – Upper quartile, Mean, SD – Standard deviation, Min – Minimal value, Max – Maximal value, NA – not available.*

|  | N | 25q | Median | 75q | Mean | SD | Min | Max | NA |
| --- | --- | --- | --- | --- | --- | --- | --- | --- | --- |
| GCL before surgery (um) | 28 | 34.47 | 41.85 | 46.28 | 39.75 | 8.09 | 22.7 | 50.2 | 0 |
| GCL 2 visit after surgery(um) | 28 | 30.45 | 41.35 | 46.50 | 38.11 | 9.46 | 20.8 | 51.5 | 0 |
| Diff. | 28 | 0.15 | 0.70 | 1.67 | 1.64 | 3.19 | -1.3 | 11.8 | 0 |


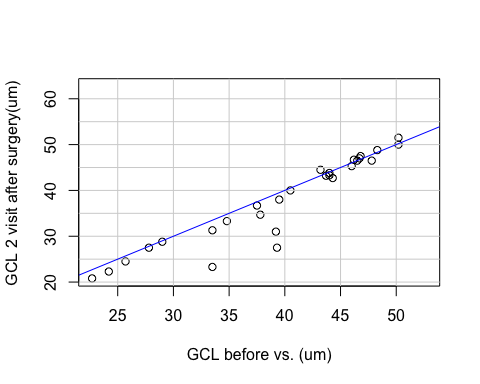


**Figure 5**Scatter plot for GCL before vs. the second visit after surgery. Each dot represents one eye, the blue link depicts the identity line.

### GCL before surgery 3 visit after surgery

*Two tail tests* Anderson-Darling test: normality = FALSE

**SIGNIFICANT** paired difference, Wilcoxon p= 0.04741625

Effect size and confidence limits, d = 0.54 [ 0.01 1.08 ]

Power of study with aforementioned effect, pwr = 0.52

GCL before surgery (um) vs. GCL 3 visit after surgery(um)

**Table 8**Descriptive statistics for the GCL before and after surgery. Parameters listed: *N – number of observations, 25q – Lower quartile, Median, 75q – Upper quartile, Mean, SD – Standard deviation, Min – Minimal value, Max – Maximal value, NA – not available.*

|  | N | 25q | Median | 75q | Mean | SD | Min | Max | NA |
| --- | --- | --- | --- | --- | --- | --- | --- | --- | --- |
| GCL before surgery (um) | 28 | 34.47 | 41.85 | 46.28 | 39.75 | 8.09 | 22.7 | 50.2 | 0 |
| GCL 3 visit after surgery(um) | 28 | 30.18 | 42.15 | 45.92 | 38.37 | 9.09 | 21.3 | 53.2 | 0 |
| Diff. | 28 | -0.32 | 0.50 | 1.73 | 1.38 | 3.53 | -4.0 | 12.1 | 0 |


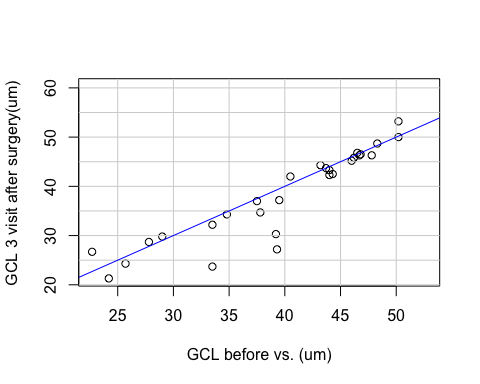


**Figure 6**Scatter plot for GCL before vs. the third visit after surgery. Each dot represents one eye, the blue link depicts the identity line.

### P100 peak time before surgery 1 visit after surgery

*Two tail tests* Anderson-Darling test: normality = FALSE

**NO** paired difference, Wilcoxon p= 0.9403642

Effect size and confidence limits, d = 0.02 [ -0.47 0.51 ]

Power of study with aforementioned effect, pwr = 0.05

P100 peak time before surgery (ms) vs. P100 peak time 1 visit after surgery(ms)

**Table 9**Descriptive statistics for the P100 peak time before and after surgery. *Parameters listed: N* – *number of observations, 25q – Lower quartile, Median, 75q* – *Upper quartile, Mean, SD – Standard deviation, Min – Minimal value, Max – Maximal value, NA – not available.*

|  | N | 25q | Median | 75q | Mean | SD | Min | Max | NA |
| --- | --- | --- | --- | --- | --- | --- | --- | --- | --- |
| P100 peak time before surgery (ms) | 32 | 98.18 | 104.55 | 117.98 | 108.89 | 14.89 | 90.0 | 145.2 | 0 |
| P100 peak time 1 visit after surgery(ms) | 32 | 99.22 | 105.60 | 115.72 | 109.99 | 15.38 | 90.6 | 153.9 | 0 |
| Diff. | 32 | -7.80 | -0.15 | 6.00 | -1.10 | 16.58 | -49.8 | 33.1 | 0 |


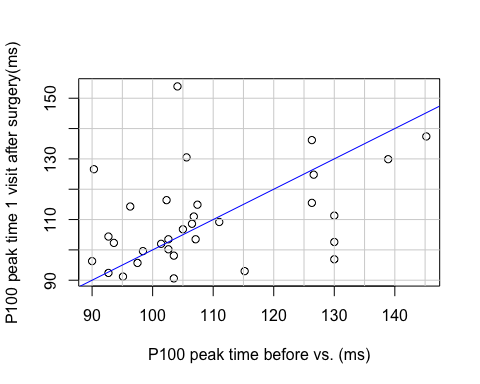


**Figure 7**Scatter plot for P100 peak time before vs. the first visit after surgery. Each dot represents one eye, the blue link depicts the identity line.

### P100 peak time before surgery 2 visit after surgery

*Two tail tests* Anderson-Darling test: normality = FALSE

**NO** paired difference, Wilcoxon p= 0.7035488

Effect size and confidence limits, d = 0.1 [ -0.41 0.61 ]

Power of study with aforementioned effect, pwr = 0.07

P100 peak time before surgery (ms) vs. P100 peak time 2 visit after surgery(ms)

**Table 10**Descriptive statistics for the P100 peak time before and after surgery. Parameters listed: *N – number of observations, 25q – Lower quartile, Median, 75q – Upper quartile, Mean, SD – Standard deviation, Min – Minimal value, Max – Maximal value, NA – not available.*

|  | N | 25q | Median | 75q | Mean | SD | Min | Max | NA |
| --- | --- | --- | --- | --- | --- | --- | --- | --- | --- |
| P100 peak time before surgery (ms) | 30 | 101.63 | 105.3 | 123.52 | 109.94 | 14.79 | 90.0 | 145.2 | 0 |
| P100 peak time 2 visit after surgery(ms) | 30 | 100.88 | 107.7 | 114.15 | 109.48 | 13.14 | 91.2 | 143.4 | 0 |
| Diff. | 30 | -2.62 | 0.6 | 10.05 | 0.46 | 18.37 | -39.3 | 35.8 | 0 |


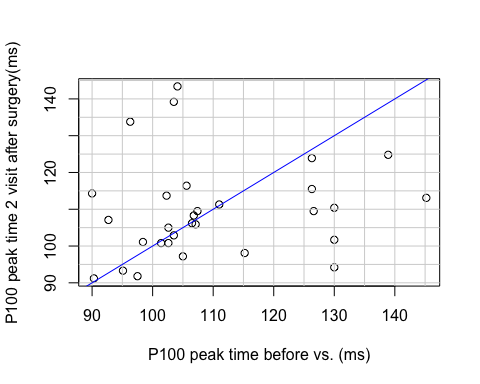


**Figure 8**Scatter plot for P100 peak time before vs. the second visit after surgery. Each dot represents one eye, the blue link depicts the identity line.

### P100 peak time before surgery 3 visit after surgery

*Two tail tests* Anderson-Darling test: normality = FALSE

**NO** paired difference, Wilcoxon p= 0.1790633

Effect size and confidence limits, d = 0.35 [ -0.16 0.86 ]

Power of study with aforementioned effect, pwr = 0.27

P100 peak time before surgery (ms) vs. P100 peak time 3 visit after surgery(ms)

**Table 11**Descriptive statistics for the P100 peak time before and after surgery. *N – number of observations, 25q – Lower quartile, Median, 75q – Upper quartile, Mean, SD – Standard deviation, Min – Minimal value, Max – Maximal value, NA – not available.*

|  | N | 25q | Median | 75q | Mean | SD | Min | Max | NA |
| --- | --- | --- | --- | --- | --- | --- | --- | --- | --- |
| P100 peak time before surgery (ms) | 30 | 101.63 | 105.30 | 123.52 | 109.94 | 14.79 | 90.0 | 145.2 | 0 |
| P100 peak time 3 visit after surgery(ms) | 30 | 99.90 | 105.15 | 111.52 | 106.63 | 12.40 | 79.2 | 142.8 | 0 |
| Diff. | 30 | -1.50 | 1.65 | 10.20 | 3.31 | 16.19 | -27.6 | 32.1 | 0 |


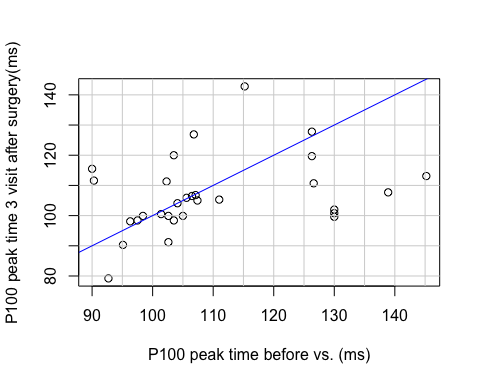


**Figure 9**Scatter plot for P100 peak time before vs. the third visit after surgery. Each dot represents one eye, the blue link depicts the identity line.

### P100 amplitude before surgery 1 visit after surgery

*Two tail tests* Anderson-Darling test: normality = TRUE

**NO** paired difference, ttest p= 0.2834693

Effect size and confidence limits, d = 0.27 [ -0.22 0.76 ]

Power of study with aforementioned effect, pwr = 0.19

P100 amplitude before surgery (uV) vs. P100 amplitude 1 visit after surgery(uV)

**Table 12**Descriptive statistics for the P100 interpeak amplitude before and after surgery. *N – number of observations, 25q – Lower quartile, Median, 75q – Upper quartile, Mean, SD – Standard deviation, Min – Minimal value, Max – Maximal value, NA – not available.*

|  | N | 25q | Median | 75q | Mean | SD | Min | Max | NA |
| --- | --- | --- | --- | --- | --- | --- | --- | --- | --- |
| P100 amplitude before surgery (uV) | 32 | 1.24 | 1.94 | 3.04 | 2.24 | 1.21 | 0.62 | 5.09 | 0 |
| P100 amplitude 1 visit after surgery(uV) | 32 | 1.76 | 2.12 | 3.06 | 2.52 | 1.21 | 0.87 | 5.59 | 0 |
| Diff. | 32 | -0.83 | -0.29 | 0.78 | -0.28 | 1.44 | -4.50 | 2.21 | 0 |


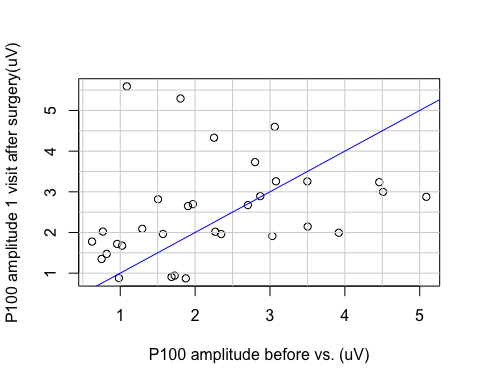


**Figure 10**Scatter plot for P100 interpeak amplitude before vs. the first visit after surgery. Each dot represents one eye, the blue link depicts the identity line.

### P100 amplitude before surgery 2 visit after surgery

*Two tail tests* Anderson-Darling test: normality = TRUE

**NO** paired difference, ttest p= 0.1036688

Effect size and confidence limits, d = 0.43 [ -0.08 0.94 ]

Power of study with aforementioned effect, pwr = 0.38

P100 amplitude before surgery (uV) vs. P100 amplitude 2 visit after surgery(uV)

**Table 13**Descriptive statistics for the P100 interpeak amplitude before and after surgery. *N – number of observations, 25q – Lower quartile, Median, 75q – Upper quartile, Mean, SD – Standard deviation, Min – Minimal value, Max – Maximal value, NA – not available.*

|  | N | 25q | Median | 75q | Mean | SD | Min | Max | NA |
| --- | --- | --- | --- | --- | --- | --- | --- | --- | --- |
| P100 amplitude before surgery (uV) | 30 | 1.14 | 1.94 | 2.99 | 2.24 | 1.23 | 0.62 | 5.09 | 0 |
| P100 amplitude 2 visit after surgery(uV) | 30 | 1.77 | 2.46 | 3.28 | 2.72 | 1.53 | 0.56 | 7.03 | 0 |
| Diff. | 30 | -0.97 | -0.19 | 0.26 | -0.49 | 1.58 | -5.10 | 1.76 | 0 |


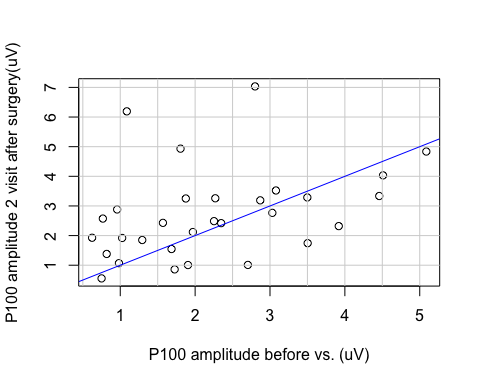


**Figure 11**Scatter plot for P100 interpeak amplitude before vs. the second visit after surgery. Each dot represents one eye, the blue link depicts the identity line.

### P100 amplitude before surgery 3 visit after surgery

*Two tail tests* Anderson-Darling test: normality = FALSE

**NO** paired difference, Wilcoxon p= 0.2449464

Effect size and confidence limits, d = 0.3 [ -0.21 0.81 ]

Power of study with aforementioned effect, pwr = 0.21

P100 amplitude before surgery (uV) vs. P100 amplitude 3 visit after surgery(uV)

**Table 14**Descriptive statistics for the P100 interpeak amplitude before and after surgery. Parameters listed: *N – number of observations, 25q – Lower quartile, Median, 75q – Upper quartile, Mean, SD – Standard deviation, Min – Minimal value, Max – Maximal value, NA – not available.*

|  | N | 25q | Median | 75q | Mean | SD | Min | Max | NA |
| --- | --- | --- | --- | --- | --- | --- | --- | --- | --- |
| P100 amplitude before surgery (uV) | 30 | 1.14 | 1.94 | 2.99 | 2.24 | 1.23 | 0.62 | 5.09 | 0 |
| P100 amplitude 3 visit after surgery(uV) | 30 | 1.75 | 2.30 | 3.03 | 2.66 | 1.43 | 0.50 | 6.16 | 0 |
| Diff. | 30 | -1.32 | -0.15 | 0.42 | -0.42 | 1.48 | -5.07 | 2.32 | 0 |


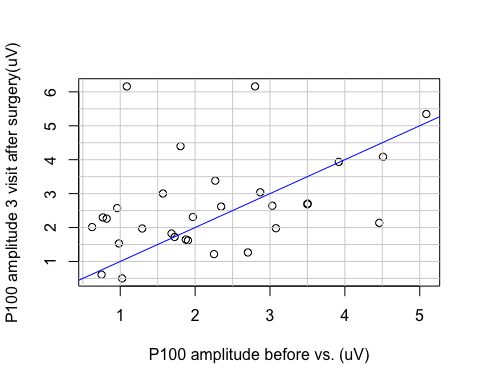


**Figure 12**Scatter plot for P100 interpeak amplitude before vs. the third visit after surgery. Each dot represents one eye, the blue link depicts the identity line.

### N160 peak time before surgery 1 visit after surgery

*Two tail tests* Anderson-Darling test: normality = TRUE

**SIGNIFICANT** paired difference, ttest p= 0.01223083

Effect size and confidence limits, d = 0.67 [ 0.15 1.19 ]

Power of study with aforementioned effect, pwr = 0.74

N160 peak time before surgery (ms) vs. N160 peak time 1 visit after surgery(ms)

**Table 15**Descriptive statistics for the N160 peak time before and after surgery. Parameters listed: *N – number of observations, 25q – Lower quartile, Median, 75q – Upper quartile, Mean, SD – Standard deviation, Min – Minimal value, Max – Maximal value, NA – not available.*

|  | N | 25q | Median | 75q | Mean | SD | Min | Max | NA |
| --- | --- | --- | --- | --- | --- | --- | --- | --- | --- |
| N160 peak time before surgery (ms) | 30 | 156.98 | 172.80 | 184.57 | 172.46 | 19.10 | 137.1 | 218.7 | 0 |
| N160 peak time 1 visit after surgery(ms) | 30 | 150.52 | 163.35 | 173.85 | 163.79 | 15.99 | 138.0 | 196.8 | 0 |
| Diff. | 30 | -1.13 | 4.50 | 19.20 | 8.67 | 17.77 | -26.1 | 48.3 | 0 |


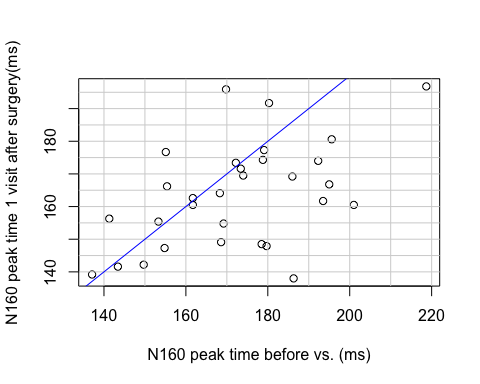


**Figure 13**Scatter plot for N160 peak time before vs. the first visit after surgery. Each dot represents one eye, the blue link depicts the identity line.

### N160 peak time before surgery 2 visit after surgery

*Two tail tests* Anderson-Darling test: normality = TRUE

**SIGNIFICANT** paired difference, ttest p= 0.00789423

Effect size and confidence limits, d = 0.74 [ 0.2 1.28 ]

Power of study with aforementioned effect, pwr = 0.79

N160 peak time before surgery (ms) vs. N160 peak time 2 visit after surgery(ms)

**Table 16**Descriptive statistics for the N160 peak time before and after surgery. *N – number of observations, 25q – Lower quartile, Median, 75q – Upper quartile, Mean, SD – Standard deviation, Min – Minimal value, Max – Maximal value, NA – not available.*

|  | N | 25q | Median | 75q | Mean | SD | Min | Max | NA |
| --- | --- | --- | --- | --- | --- | --- | --- | --- | --- |
| N160 peak time before surgery (ms) | 28 | 161.70 | 173.7 | 186.07 | 174.76 | 17.57 | 141.3 | 218.7 | 0 |
| N160 peak time 2 visit after surgery(ms) | 28 | 156.75 | 168.3 | 175.35 | 166.10 | 11.05 | 146.1 | 185.4 | 0 |
| Diff. | 28 | 1.20 | 8.7 | 17.77 | 8.66 | 15.96 | -39.0 | 35.7 | 0 |


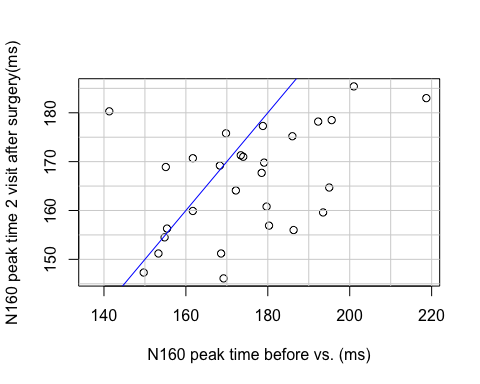


**Figure 14**Scatter plot for N160 peak time before vs. the second visit after surgery. Each dot represents one eye, the blue link depicts the identity line.

### N160 peak time before surgery 3 visit after surgery

*Two tail tests* Anderson-Darling test: normality = TRUE

**SIGNIFICANT** paired difference, ttest p= 0.01517973

Effect size and confidence limits, d = 0.67 [ 0.13 1.21 ]

Power of study with aforementioned effect, pwr = 0.71

N160 peak time before surgery (ms) vs. N160 peak time 3 visit after surgery(ms)

**Table 17**Descriptive statistics for the N160 peak time before and after surgery. Parameters listed: *N – number of observations, 25q – Lower quartile, Median, 75q – Upper quartile, Mean, SD – Standard deviation, Min – Minimal value, Max – Maximal value, NA – not available.*

|  | N | 25q | Median | 75q | Mean | SD | Min | Max | NA |
| --- | --- | --- | --- | --- | --- | --- | --- | --- | --- |
| N160 peak time before surgery (ms) | 28 | 161.70 | 173.70 | 186.07 | 174.76 | 17.57 | 141.3 | 218.7 | 0 |
| N160 peak time 3 visit after surgery(ms) | 28 | 156.68 | 166.35 | 176.85 | 165.55 | 11.97 | 144.3 | 191.1 | 0 |
| Diff. | 28 | -4.72 | 9.60 | 18.30 | 9.21 | 18.80 | -24.3 | 49.2 | 0 |


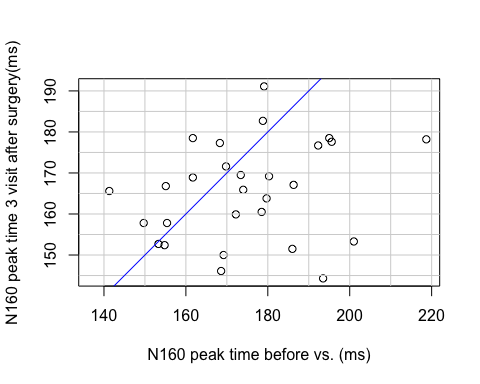


**Figure 15**Scatter plot for N160 peak time before vs. the third visit after surgery. Each dot represents one eye, the blue link depicts the identity line.

### N160 amplitude before surgery 1 visit after surgery

*Two tail tests* Anderson-Darling test: normality = TRUE

**NO** paired difference, ttest p= 0.2895562

Effect size and confidence limits, d = 0.28 [ -0.23 0.78 ]

Power of study with aforementioned effect, pwr = 0.19

N160 amplitude before surgery (uV) vs. N160 amplitude 1 visit after surgery(uV)

**Table 18**Descriptive statistics for the N160 interpeak amplitude before and after surgery. Parameters listed: *N – number of observations, 25q – Lower quartile, Median, 75q – Upper quartile, Mean, SD – Standard deviation, Min – Minimal value, Max – Maximal value, NA – not available.*

|  | N | 25q | Median | 75q | Mean | SD | Min | Max | NA |
| --- | --- | --- | --- | --- | --- | --- | --- | --- | --- |
| N160 amplitude before surgery (uV) | 30 | 2.70 | 4.01 | 5.05 | 3.86 | 1.48 | 1.18 | 6.95 | 0 |
| N160 amplitude 1 visit after surgery(uV) | 30 | 3.77 | 4.42 | 5.22 | 4.25 | 1.45 | 0.91 | 6.48 | 0 |
| Diff. | 30 | -1.06 | -0.44 | 0.19 | -0.38 | 1.95 | -4.81 | 5.76 | 0 |


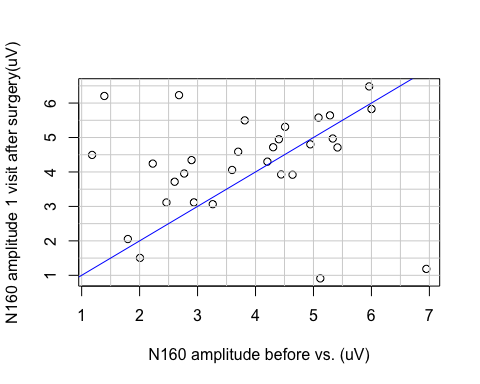


**Figure 16**Scatter plot for N160 interpeak amplitude before vs. the first visit after surgery. Each dot represents one eye, the blue link depicts the identity line.

### N160 amplitude before surgery 2 visit after surgery

*Two tail tests* Anderson-Darling test: normality = TRUE

**SIGNIFICANT** paired difference, ttest p= 0.03416781

Effect size and confidence limits, d = 0.58 [ 0.05 1.12 ]

Power of study with aforementioned effect, pwr = 0.58

N160 amplitude before surgery (uV) vs. N160 amplitude 2 visit after surgery(uV)

**Table 19**Descriptive statistics for the N160 interpeak amplitude before and after surgery. Parameters listed: *N – number of observations, 25q – Lower quartile, Median, 75q – Upper quartile, Mean, SD – Standard deviation, Min – Minimal value, Max – Maximal value, NA – not available.*

|  | N | 25q | Median | 75q | Mean | SD | Min | Max | NA |
| --- | --- | --- | --- | --- | --- | --- | --- | --- | --- |
| N160 amplitude before surgery (uV) | 28 | 2.66 | 4.01 | 4.98 | 3.82 | 1.46 | 1.18 | 6.95 | 0 |
| N160 amplitude 2 visit after surgery(uV) | 28 | 2.93 | 4.26 | 5.11 | 4.26 | 1.48 | 1.32 | 7.78 | 0 |
| Diff. | 28 | -0.73 | -0.21 | 0.18 | -0.44 | 1.03 | -3.39 | 1.13 | 0 |


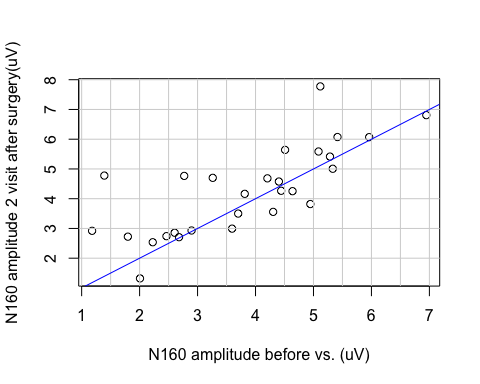


**Figure 17**Scatter plot for N160 interpeak amplitude before vs. the second visit after surgery. Each dot represents one eye, the blue link depicts the identity line.

### N160 amplitude before surgery 3 visit after surgery

*Two tail tests* Anderson-Darling test: normality = TRUE

**NO** paired difference, ttest p= 0.3463546

Effect size and confidence limits, d = 0.25 [ -0.27 0.78 ]

Power of study with aforementioned effect, pwr = 0.15

N160 amplitude before surgery (uV) vs. N160 amplitude 3 visit after surgery(uV)

**Table 20**Descriptive statistics for the N160 interpeak amplitude before and after surgery. Parameters listed: *N – number of observations, 25q – Lower quartile, Median, 75q – Upper quartile, Mean, SD – Standard deviation, Min – Minimal value, Max – Maximal value, NA – not available.*

|  | N | 25q | Median | 75q | Mean | SD | Min | Max | NA |
| --- | --- | --- | --- | --- | --- | --- | --- | --- | --- |
| N160 amplitude before surgery (uV) | 28 | 2.66 | 4.01 | 4.98 | 3.82 | 1.46 | 1.18 | 6.95 | 0 |
| N160 amplitude 3 visit after surgery(uV) | 28 | 2.98 | 3.91 | 5.12 | 4.19 | 1.54 | 1.52 | 7.53 | 0 |
| Diff. | 28 | -1.53 | -0.26 | 0.68 | -0.37 | 2.02 | -5.47 | 4.66 | 0 |


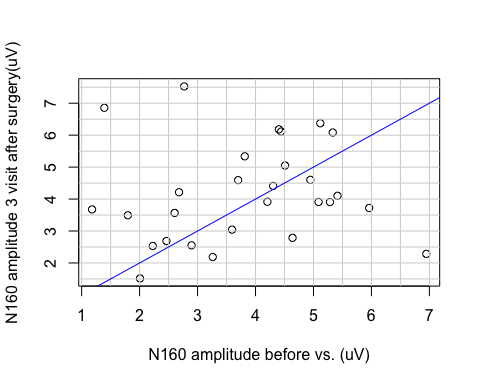


**Figure 18**Scatter plot for N160 interpeak amplitude before vs. the third visit after surgery. Each dot represents one eye, the blue link depicts the identity line.

### Visual acuity before surgery 1 visit after surgery

*Two tail tests* Anderson-Darling test: normality = FALSE

**SIGNIFICANT** paired difference, Wilcoxon p= 0.01009348

Effect size and confidence limits, d = 0.66 [ 0.16 1.17 ]

Power of study with aforementioned effect, pwr = 0.75

Visual acuity before surgery (logMAR) vs. Visual acuity 1 visit after surgery(logMAR)

**Table 21**Descriptive statistics for the visual acuity before and after surgery. Parameters listed: *N – number of observations, 25q – Lower quartile, Median, 75q – Upper quartile, Mean, SD – Standard deviation, Min – Minimal value, Max – Maximal value, NA – not available.*

|  | N | 25q | Median | 75q | Mean | SD | Min | Max | NA |
| --- | --- | --- | --- | --- | --- | --- | --- | --- | --- |
| Visual acuity before surgery (logMAR) | 32 | 0.1 | 0.40 | 0.72 | 0.48 | 0.47 | -0.20 | 1.4 | 0 |
| Visual acuity 1 visit after surgery(logMAR) | 32 | 0.1 | 0.30 | 0.63 | 0.36 | 0.33 | -0.12 | 1.0 | 0 |
| Diff. | 32 | 0.0 | 0.09 | 0.24 | 0.12 | 0.26 | -0.51 | 0.7 | 0 |


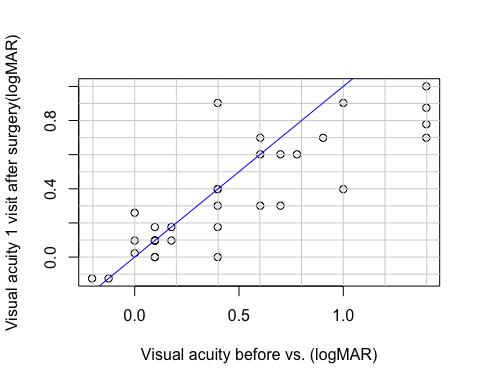


**Figure 19**Scatter plot for visual acuity before vs. the first visit after surgery. Each dot represents one eye, the blue link depicts the identity line.

### Visual acuity before surgery 2 visit after surgery

*Two tail tests* Anderson-Darling test: normality = FALSE

**SIGNIFICANT** paired difference, Wilcoxon p= 9.790719e-06

Effect size and confidence limits, d = 1.25 [ 0.7 1.8 ]

Power of study with aforementioned effect, pwr = 1

Visual acuity before surgery (logMAR) vs. Visual acuity 2 visit after surgery(logMAR)

**Table 22**Descriptive statistics for the visual acuity before and after surgery. Parameters listed: *N – number of observations, 25q – Lower quartile, Median, 75q – Upper quartile, Mean, SD – Standard deviation, Min – Minimal value, Max – Maximal value, NA – not available.*

|  | N | 25q | Median | 75q | Mean | SD | Min | Max | NA |
| --- | --- | --- | --- | --- | --- | --- | --- | --- | --- |
| Visual acuity before surgery (logMAR) | 30 | 0.1 | 0.40 | 0.76 | 0.51 | 0.48 | -0.20 | 1.40 | 0 |
| Visual acuity 2 visit after surgery(logMAR) | 30 | 0.0 | 0.30 | 0.40 | 0.24 | 0.29 | -0.12 | 1.00 | 0 |
| Diff. | 30 | 0.1 | 0.15 | 0.36 | 0.27 | 0.30 | -0.08 | 1.22 | 0 |


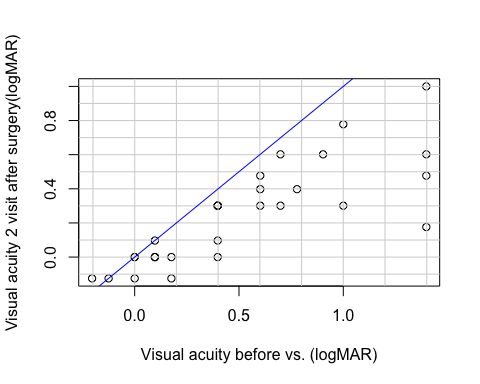


**Figure 20**Scatter plot for visual acuity before vs. the second visit after surgery. Each dot represents one eye, the blue link depicts the identity line.

### Visual acuity before surgery 3 visit after surgery

*Two tail tests* Anderson-Darling test: normality = TRUE

**SIGNIFICANT** paired difference, ttest p= 3.167806e-05

Effect size and confidence limits, d = 1.21 [ 0.64 1.78 ]

Power of study with aforementioned effect, pwr = 0.99

Visual acuity before surgery (logMAR) vs. Visual acuity 3 visit after surgery(logMAR)

**Table 23**Descriptive statistics for the visual acuity before and after surgery. Parameters listed: *N – number of observations, 25q – Lower quartile, Median, 75q – Upper quartile, Mean, SD – Standard deviation, Min – Minimal value, Max – Maximal value, NA – not available.*

|  | N | 25q | Median | 75q | Mean | SD | Min | Max | NA |
| --- | --- | --- | --- | --- | --- | --- | --- | --- | --- |
| Visual acuity before surgery (logMAR) | 28 | 0.1 | 0.40 | 0.81 | 0.54 | 0.48 | -0.2 | 1.40 | 0 |
| Visual acuity 3 visit after surgery(logMAR) | 28 | 0.1 | 0.24 | 0.40 | 0.25 | 0.25 | -0.2 | 0.90 | 0 |
| Diff. | 28 | 0.0 | 0.22 | 0.42 | 0.29 | 0.31 | -0.1 | 1.22 | 0 |


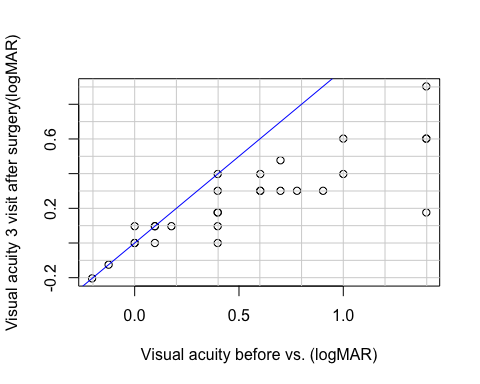


**Figure 21**Scatter plot for visual acuity before vs. the third visit after surgery. Each dot represents one eye, the blue link depicts the identity line.

### Perimetry before surgery 1 visit after surgery

*Two tail tests* Anderson-Darling test: normality = FALSE

**SIGNIFICANT** paired difference, Wilcoxon p= 1.259148e-06

Effect size and confidence limits, d = 1.46 [ 0.87 2.05 ]

Power of study with aforementioned effect, pwr = 1

Perimetry before surgery (MD) vs. Perimetry 1 visit after surgery(MD)

**Table 24**Descriptive statistics for the perimetry before and after surgery. Parameters listed: *N – number of observations, 25q – Lower quartile, Median, 75q – Upper quartile, Mean, SD – Standard deviation, Min – Minimal value, Max – Maximal value, NA – not available.*

|  | N | 25q | Median | 75q | Mean | SD | Min | Max | NA |
| --- | --- | --- | --- | --- | --- | --- | --- | --- | --- |
| Perimetry before surgery (MD) | 28 | -14.25 | -4.82 | -1.84 | -8.69 | 8.32 | -27.18 | 0.02 | 0 |
| Perimetry 1 visit after surgery(MD) | 28 | -6.06 | -2.68 | -0.79 | -4.77 | 5.68 | -22.79 | 0.92 | 0 |
| Diff. | 28 | -4.60 | -2.32 | -0.54 | -3.92 | 5.80 | -24.27 | 2.57 | 0 |


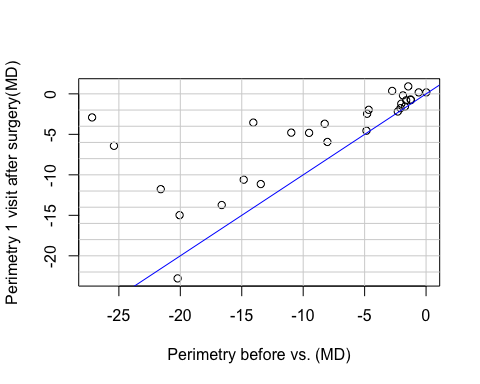


**Figure 22**Scatter plot for perimetry before vs. the first visit after surgery. Each dot represents one eye, the blue link depicts the identity line.

### Perimetry before surgery 2 visit after surgery

*Two tail tests* Anderson-Darling test: normality = FALSE

**SIGNIFICANT** paired difference, Wilcoxon p= 3.539026e-08

Effect size and confidence limits, d = 1.64 [ 1.06 2.23 ]

Power of study with aforementioned effect, pwr = 1

Perimetry before surgery (MD) vs. Perimetry 2 visit after surgery(MD)

**Table 25**Descriptive statistics for the perimetry before and after surgery. Parameters listed: *N – number of observations, 25q – Lower quartile, Median, 75q – Upper quartile, Mean, SD – Standard deviation, Min – Minimal value, Max – Maximal value, NA – not available.*

|  | N | 25q | Median | 75q | Mean | SD | Min | Max | NA |
| --- | --- | --- | --- | --- | --- | --- | --- | --- | --- |
| Perimetry before surgery (MD) | 30 | -13.91 | -4.73 | -1.69 | -8.24 | 8.23 | -27.18 | 0.02 | 0 |
| Perimetry 2 visit after surgery(MD) | 30 | -3.40 | -0.99 | -0.37 | -2.63 | 3.90 | -18.35 | 0.50 | 0 |
| Diff. | 30 | -8.56 | -2.48 | -1.14 | -5.60 | 6.88 | -26.35 | 0.65 | 0 |


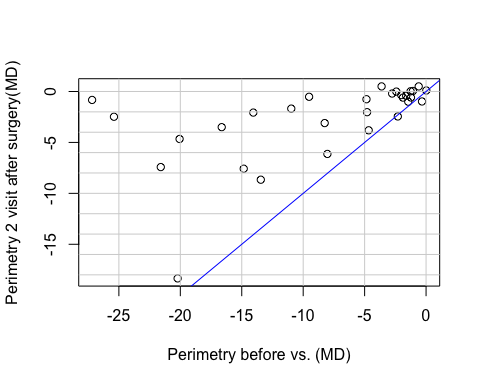


**Figure 23**Scatter plot for perimetry before vs. the second visit after surgery. Each dot represents one eye, the blue link depicts the identity line.

### Perimetry before surgery 3 visit after surgery

*Two tail tests* Anderson-Darling test: normality = FALSE

**SIGNIFICANT** paired difference, Wilcoxon p= 2.607703e-08

Effect size and confidence limits, d = 1.66 [ 1.07 2.25 ]

Power of study with aforementioned effect, pwr = 1

Perimetry before surgery (MD) vs. Perimetry 3 visit after surgery(MD)

**Table 26**Descriptive statistics for the perimetry before and after surgery. Parameters listed: *N – number of observations, 25q – Lower quartile, Median, 75q – Upper quartile, Mean, SD – Standard deviation, Min – Minimal value, Max – Maximal value, NA – not available.*

|  | N | 25q | Median | 75q | Mean | SD | Min | Max | NA |
| --- | --- | --- | --- | --- | --- | --- | --- | --- | --- |
| Perimetry before surgery (MD) | 30 | -13.91 | -4.73 | -1.69 | -8.24 | 8.23 | -27.18 | 0.02 | 0 |
| Perimetry 3 visit after surgery(MD) | 30 | -2.93 | -1.17 | 0.21 | -2.07 | 4.95 | -19.04 | 11.83 | 0 |
| Diff. | 30 | -9.28 | -2.50 | -0.96 | -6.16 | 7.33 | -25.82 | 0.64 | 0 |


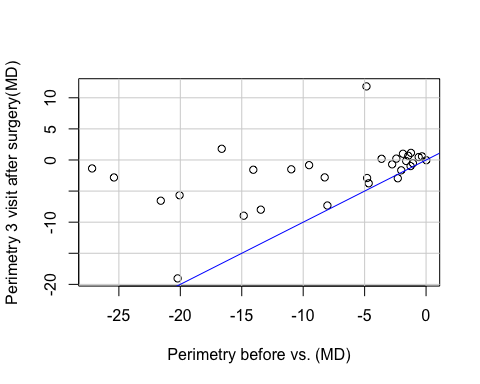


**Figure 24**Scatter plot for perimetry before vs. the third visit after surgery. Each dot represents one eye, the blue link depicts the identity line.

# Grade

### RNFL

**Table 27**Descriptive statistics for the RNFL grouped by combination of the grade and the visit factors. Parameters listed: N – number of observations, 25q – Lower quartile, Median, 75q – Upper quartile, Mean, SD – Standard deviation, Min – Minimal value, Max – Maximal value, NA – not available.

|  | N | 25q | Median | 75q | Mean | SD | Min | Max | NA |
| --- | --- | --- | --- | --- | --- | --- | --- | --- | --- |
| Pre.lowG | 8 | 69.75 | 76.5 | 84.75 | 80.000 | 15.547 | 62 | 105 | 2 |
| X1st.lowG | 10 | 71.25 | 76.0 | 81.00 | 79.900 | 13.988 | 63 | 106 | 0 |
| X2nd.lowG | 10 | 70.00 | 76.5 | 82.00 | 79.300 | 12.936 | 65 | 104 | 0 |
| X3rd.lowG | 10 | 69.00 | 76.0 | 81.00 | 79.200 | 14.589 | 63 | 109 | 0 |
| Pre.hiG | 22 | 46.00 | 61.0 | 66.75 | 58.091 | 15.433 | 27 | 84 | 0 |
| X1st.hiG | 22 | 44.25 | 59.5 | 67.75 | 57.955 | 15.756 | 27 | 84 | 0 |
| X2nd.hiG | 20 | 38.75 | 54.0 | 65.00 | 53.150 | 17.670 | 23 | 83 | 2 |
| X3rd.hiG | 20 | 37.25 | 52.0 | 64.25 | 52.250 | 17.363 | 23 | 83 | 2 |

Anderson-Darling test for normality

Bartlett test of variances homogeneity p = 0.9689196

Normality accepted = * FALSE *

**SIGNIFICANT** difference between groups, p= 1.797995e-07

**Table 28**Wilcoxon rank sum test with continuity correction

|  | Pre.lowG | 1st.lowG | 2nd.lowG | 3rd.lowG | Pre.hiG | 1st.hiG | 2nd.hiG |
| --- | --- | --- | --- | --- | --- | --- | --- |
| 1st.lowG | 1.0000000 | NA | NA | NA | NA | NA | NA |
| 2nd.lowG | 1.0000000 | 1.0000000 | NA | NA | NA | NA | NA |
| 3rd.lowG | 1.0000000 | 1.0000000 | 1.0000000 | NA | NA | NA | NA |
| Pre.hiG | 0.0545542 | 0.0254829 | 0.0254829 | 0.0312590 | NA | NA | NA |
| 1st.hiG | 0.0465923 | 0.0229683 | 0.0254829 | 0.0336247 | 1 | NA | NA |
| 2nd.hiG | 0.0368338 | 0.0229683 | 0.0186767 | 0.0229683 | 1 | 1 | NA |
| 3rd.hiG | 0.0336247 | 0.0196201 | 0.0129765 | 0.0196201 | 1 | 1 | 1 |


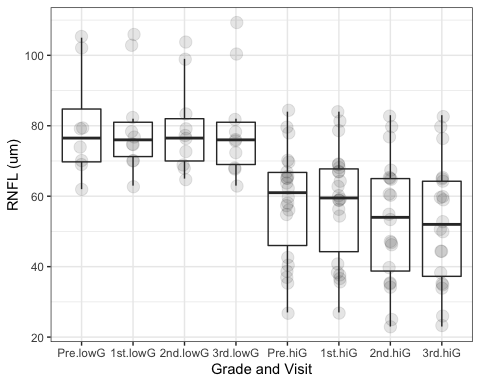


**Figure 25**Box chart plotting RNFL for combinations of the grade and the visit factors. Each dot represents one eye, the box depicts the medina and upper and lower quartile.

### GCL

**Table 29**Descriptive statistics for the GCL grouped by combination of the grade and the visit factors. Parameters listed N – number of observations, 25q – Lower quartile, Median, 75q – Upper quartile, Mean, SD – Standard deviation, Min – Minimal value, Max – Maximal value, NA – not available.

|  | N | 25q | Median | 75q | Mean | SD | Min | Max | NA |
| --- | --- | --- | --- | --- | --- | --- | --- | --- | --- |
| Pre.lowG | 8 | 43.925 | 46.25 | 48.775 | 46.175 | 3.383 | 40.5 | 50.2 | 2 |
| X1st.lowG | 10 | 44.100 | 47.50 | 50.100 | 48.910 | 6.584 | 42.3 | 61.8 | 0 |
| X2nd.lowG | 10 | 43.800 | 47.65 | 51.125 | 49.150 | 7.358 | 40.0 | 62.7 | 0 |
| X3rd.lowG | 10 | 44.075 | 47.75 | 52.400 | 49.070 | 6.480 | 42.0 | 60.3 | 0 |
| Pre.hiG | 22 | 33.500 | 39.25 | 45.725 | 38.532 | 8.806 | 22.7 | 52.7 | 0 |
| X1st.hiG | 22 | 33.275 | 38.65 | 44.475 | 38.109 | 8.282 | 23.3 | 52.3 | 0 |
| X2nd.hiG | 20 | 27.500 | 34.00 | 43.975 | 34.920 | 9.182 | 20.8 | 47.5 | 2 |
| X3rd.hiG | 20 | 28.325 | 34.50 | 43.550 | 35.120 | 8.526 | 21.3 | 46.5 | 2 |

Anderson-Darling test for normality

Bartlett test of variances homogeneity p = 0.2637149

Normality accepted = * TRUE *

**SIGNIFICANT** difference between groups, anova p= 2.357888e-08

***Table 30***T-tests with pooled SD

|  | Pre.lowG | 1st.lowG | 2nd.lowG | 3rd.lowG | Pre.hiG | 1st.hiG | 2nd.hiG |
| --- | --- | --- | --- | --- | --- | --- | --- |
| 1st.lowG | 1.0000000 | NA | NA | NA | NA | NA | NA |
| 2nd.lowG | 1.0000000 | 1.0000000 | NA | NA | NA | NA | NA |
| 3rd.lowG | 1.0000000 | 1.0000000 | 1.0000000 | NA | NA | NA | NA |
| Pre.hiG | 0.3027050 | 0.0168646 | 0.0145282 | 0.0150182 | NA | NA | NA |
| 1st.hiG | 0.2351235 | 0.0125087 | 0.0105299 | 0.0109936 | 1 | NA | NA |
| 2nd.hiG | 0.0179970 | 0.0004334 | 0.0003566 | 0.0003812 | 1 | 1 | NA |
| 3rd.hiG | 0.0204740 | 0.0005142 | 0.0004282 | 0.0004378 | 1 | 1 | 1 |


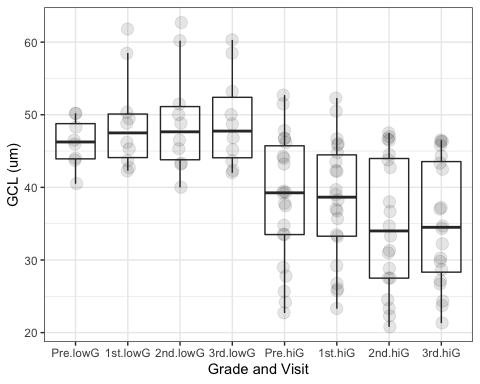


**Figure 26**Box chart plotting GCL for combinations of the grade and the visit factors. Each dot represents one eye, the box depicts the medina and upper and lower quartile.

### P100 peak time

**Table 31**Descriptive statistics for the P100 Peak time grouped by combination of the grade and the visit factors. Parameters listed: N – number of observations, 25q – Lower quartile, Median, 75q – Upper quartile, Mean, SD – Standard deviation, Min – Minimal value, Max – Maximal value, NA – not available.

|  | N | 25q | Median | 75q | Mean | SD | Min | Max | NA |
| --- | --- | --- | --- | --- | --- | --- | --- | --- | --- |
| Pre.lowG | 10 | 99.150 | 102.45 | 105.750 | 102.120 | 4.217 | 95.1 | 107.4 | 0 |
| X1st.lowG | 10 | 99.750 | 102.75 | 109.125 | 103.800 | 8.112 | 91.2 | 116.4 | 0 |
| X2nd.lowG | 10 | 100.800 | 103.05 | 107.700 | 103.020 | 6.913 | 91.8 | 113.7 | 0 |
| X3rd.lowG | 10 | 98.775 | 100.20 | 106.350 | 103.020 | 10.571 | 90.3 | 126.9 | 0 |
| Pre.hiG | 22 | 98.100 | 106.05 | 126.525 | 111.968 | 16.964 | 90.0 | 145.2 | 0 |
| X1st.hiG | 22 | 99.150 | 108.90 | 126.150 | 112.800 | 17.165 | 90.6 | 153.9 | 0 |
| X2nd.hiG | 20 | 102.600 | 110.85 | 118.275 | 112.710 | 14.422 | 91.2 | 143.4 | 2 |
| X3rd.hiG | 20 | 100.575 | 106.20 | 113.700 | 108.435 | 13.090 | 79.2 | 142.8 | 2 |

Anderson-Darling test for normality

Bartlett test of variances homogeneity p = 0.000288476

Normality accepted = * FALSE *

**NO** intergroup difference, Kruskal-Wallis p= 0.2101178


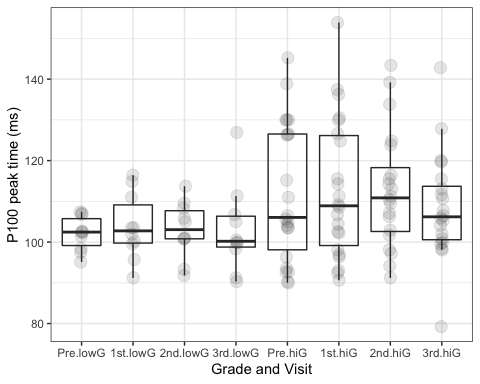


**Figure 27**Box chart plotting P100 peak time for combinations of the grade and the visit factors. Each dot represents one eye, the box depicts the medina and upper and lower quartile.

### P100 amplitude

**Table 32**Descriptive statistics for the P100 interpeak amplitude grouped by combination of the grade and the visit factors. Parameters listed: N – number of observations, 25q – Lower quartile, Median, 75q – Upper quartile, Mean, SD – Standard deviation, Min – Minimal value, Max – Maximal value, NA – not available.

|  | N | 25q | Median | 75q | Mean | SD | Min | Max | NA |
| --- | --- | --- | --- | --- | --- | --- | --- | --- | --- |
| Pre.lowG | 10 | 2.276 | 2.868 | 3.502 | 3.013 | 1.170 | 1.294 | 5.088 | 0 |
| X1st.lowG | 10 | 2.104 | 2.662 | 2.968 | 2.690 | 0.741 | 1.914 | 4.331 | 0 |
| X2nd.lowG | 10 | 1.771 | 2.454 | 3.157 | 2.544 | 1.244 | 1.006 | 4.836 | 0 |
| X3rd.lowG | 10 | 1.708 | 2.631 | 2.702 | 2.616 | 1.283 | 1.217 | 5.348 | 0 |
| Pre.hiG | 22 | 0.993 | 1.706 | 2.668 | 1.891 | 1.073 | 0.624 | 4.459 | 0 |
| X1st.hiG | 22 | 1.525 | 2.006 | 3.151 | 2.441 | 1.381 | 0.871 | 5.592 | 0 |
| X2nd.hiG | 20 | 1.826 | 2.501 | 3.278 | 2.815 | 1.673 | 0.555 | 7.034 | 2 |
| X3rd.hiG | 20 | 1.799 | 2.280 | 3.125 | 2.675 | 1.526 | 0.501 | 6.161 | 2 |

Anderson-Darling test for normality

Bartlett test of variances homogeneity p = 0.2323035

Normality accepted = * FALSE *

**NO** intergroup difference, Kruskal-Wallis p= 0.1950085


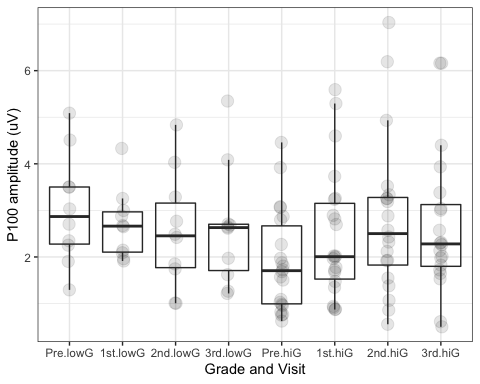


**Figure 28**Box chart plotting P100 interpeak amplitude for combinations of the grade and the visit factors. Each dot represents one eye, the box depicts the medina and upper and lower quartile.

### N160 peak time

**Table 33**Descriptive statistics for the N160 peak time grouped by combination of the grade and the visit factors. Parameters listed: N – number of observations, 25q – Lower quartile, Median, 75q – Upper quartile, Mean, SD – Standard deviation, Min – Minimal value, Max – Maximal value, NA – not available.

|  | N | 25q | Median | 75q | Mean | SD | Min | Max | NA |
| --- | --- | --- | --- | --- | --- | --- | --- | --- | --- |
| Pre.lowG | 10 | 164.325 | 175.95 | 179.550 | 173.370 | 10.716 | 155.1 | 186.3 | 0 |
| X1st.lowG | 10 | 151.500 | 165.90 | 172.950 | 162.570 | 13.665 | 138.0 | 177.3 | 0 |
| X2nd.lowG | 10 | 161.625 | 168.30 | 170.475 | 166.440 | 6.023 | 156.0 | 175.2 | 0 |
| X3rd.lowG | 10 | 161.325 | 166.95 | 169.350 | 167.760 | 10.854 | 151.5 | 191.1 | 0 |
| Pre.hiG | 20 | 154.425 | 169.50 | 192.600 | 172.005 | 22.403 | 137.1 | 218.7 | 2 |
| X1st.hiG | 20 | 153.375 | 162.90 | 174.075 | 164.400 | 17.336 | 139.2 | 196.8 | 2 |
| X2nd.hiG | 18 | 154.950 | 166.95 | 177.975 | 165.917 | 13.216 | 146.1 | 185.4 | 4 |
| X3rd.hiG | 18 | 152.850 | 165.75 | 177.150 | 164.317 | 12.677 | 144.3 | 182.7 | 4 |

Anderson-Darling test for normality

Bartlett test of variances homogeneity p = 0.002692955

Normality accepted = * FALSE *

**NO** intergroup difference, Kruskal-Wallis p= 0.5786729


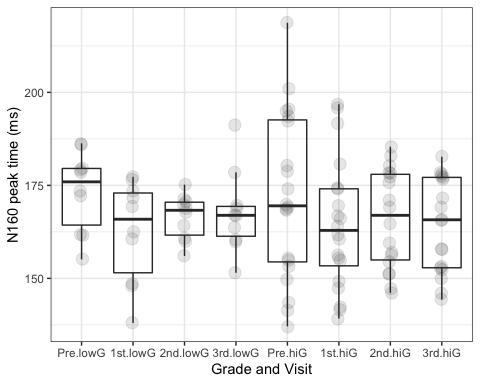


**Figure 29**Box chart plotting N160 peak time for combinations of the grade and the visit factors. Each dot represents one eye, the box depicts the medina and upper and lower quartile.

### N160 amplitude

**Table 34**Descriptive statistics for the N160 interpeak amplitude grouped by combination of the grade and the visit factors. Parameters listed: N – number of observations, 25q – Lower quartile, Median, 75q – Upper quartile, Mean, SD – Standard deviation, Min – Minimal value, Max – Maximal value, NA – not available.

|  | N | 25q | Median | 75q | Mean | SD | Min | Max | NA |
| --- | --- | --- | --- | --- | --- | --- | --- | --- | --- |
| Pre.lowG | 10 | 3.098 | 4.059 | 5.271 | 4.134 | 1.297 | 2.227 | 5.961 | 0 |
| X1st.lowG | 10 | 4.405 | 4.715 | 5.366 | 4.885 | 0.791 | 3.716 | 6.484 | 0 |
| X2nd.lowG | 10 | 3.073 | 3.860 | 5.442 | 4.228 | 1.360 | 2.537 | 6.073 | 0 |
| X3rd.lowG | 10 | 3.605 | 4.006 | 4.547 | 4.081 | 1.111 | 2.532 | 6.083 | 0 |
| Pre.hiG | 20 | 2.626 | 3.900 | 4.715 | 3.729 | 1.575 | 1.180 | 6.947 | 2 |
| X1st.hiG | 20 | 3.102 | 4.006 | 5.040 | 3.929 | 1.611 | 0.911 | 6.230 | 2 |
| X2nd.hiG | 18 | 2.938 | 4.421 | 4.775 | 4.272 | 1.579 | 1.321 | 7.775 | 4 |
| X3rd.hiG | 18 | 2.851 | 3.911 | 5.847 | 4.245 | 1.762 | 1.517 | 7.526 | 4 |

Anderson-Darling test for normality

Bartlett test of variances homogeneity p = 0.3349135

Normality accepted = * TRUE *

**NO** intergroup difference, anova p= 0.6960412


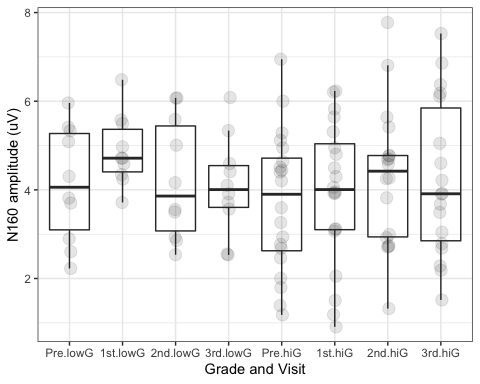


**Figure 30**Box chart plotting N160 interpeak amplitude for combinations of the grade and the visit factors. Each dot represents one eye, the box depicts the medina and upper and lower quartile.

### Visual acuity

**Table 35**Descriptive statistics for the visual acuity grouped by combination of the grade and the visit factors. Parameters listed: N – number of observations, 25q – Lower quartile, Median, 75q – Upper quartile, Mean, SD – Standard deviation, Min – Minimal value, Max – Maximal value, NA – not available.

|  | N | 25q | Median | 75q | Mean | SD | Min | Max | NA |
| --- | --- | --- | --- | --- | --- | --- | --- | --- | --- |
| Pre.lowG | 10 | 0.000 | 0.048 | 0.323 | 0.136 | 0.278 | -0.204 | 0.699 | 0 |
| X1st.lowG | 10 | 0.041 | 0.137 | 0.239 | 0.128 | 0.172 | -0.125 | 0.398 | 0 |
| X2nd.lowG | 10 | -0.094 | 0.000 | 0.073 | 0.032 | 0.159 | -0.125 | 0.301 | 0 |
| X3rd.lowG | 10 | 0.000 | 0.097 | 0.097 | 0.054 | 0.144 | -0.204 | 0.301 | 0 |
| Pre.hiG | 22 | 0.232 | 0.602 | 0.976 | 0.641 | 0.463 | 0.097 | 1.398 | 0 |
| X1st.hiG | 22 | 0.117 | 0.500 | 0.699 | 0.465 | 0.335 | 0.000 | 1.000 | 0 |
| X2nd.hiG | 20 | 0.156 | 0.301 | 0.508 | 0.349 | 0.284 | -0.125 | 1.000 | 2 |
| X3rd.hiG | 18 | 0.207 | 0.301 | 0.457 | 0.352 | 0.230 | 0.000 | 0.903 | 4 |

Anderson-Darling test for normality

Bartlett test of variances homogeneity p = 0.0002388924

Normality accepted = * FALSE *

**SIGNIFICANT** difference between groups, p= 1.420894e-06

**Table 36**Wilcoxon rank sum test with continuity correction

|  | Pre.lowG | 1st.lowG | 2nd.lowG | 3rd.lowG | Pre.hiG | 1st.hiG | 2nd.hiG |
| --- | --- | --- | --- | --- | --- | --- | --- |
| 1st.lowG | 1.0000000 | NA | NA | NA | NA | NA | NA |
| 2nd.lowG | 1.0000000 | 1.0000000 | NA | NA | NA | NA | NA |
| 3rd.lowG | 1.0000000 | 1.0000000 | 1.0000000 | NA | NA | NA | NA |
| Pre.hiG | 0.0421736 | 0.0436574 | 0.0026436 | 0.0054132 | NA | NA | NA |
| 1st.hiG | 0.2377255 | 0.2475372 | 0.0173538 | 0.0506911 | 1.0000000 | NA | NA |
| 2nd.hiG | 0.7960138 | 0.5026052 | 0.0598483 | 0.1121392 | 0.5810918 | 1 | NA |
| 3rd.hiG | 0.5810918 | 0.2475372 | 0.0234720 | 0.0331735 | 0.6027325 | 1 | 1 |


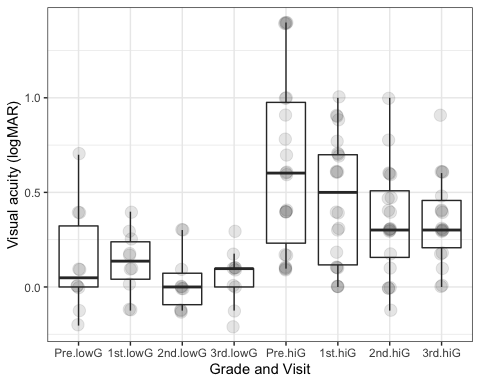


**Figure 31**Box chart plotting visual acuity for combinations of the grade and the visit factors. Each dot represents one eye, the box depicts the medina and upper and lower quartile.

### Perimetry

**Table 37**Descriptive statistics for the perimetry grouped by combination of the grade and the visit factors. Parameters listed: N – number of observations, 25q – Lower quartile, Median, 75q – Upper quartile, Mean, SD – Standard deviation, Min – Minimal value, Max – Maximal value, NA – not available.

|  | N | 25q | Median | 75q | Mean | SD | Min | Max | NA |
| --- | --- | --- | --- | --- | --- | --- | --- | --- | --- |
| Pre.lowG | 10 | -3.320 | -1.535 | -1.110 | -2.504 | 2.367 | -8.03 | -0.33 | 0 |
| X1st.lowG | 6 | -1.678 | -0.785 | -0.057 | -1.397 | 2.432 | -5.94 | 0.92 | 4 |
| X2nd.lowG | 10 | -0.995 | -0.505 | 0.035 | -1.189 | 2.133 | -6.13 | 0.50 | 0 |
| X3rd.lowG | 10 | -0.438 | 0.205 | 0.552 | -0.849 | 2.649 | -7.32 | 1.14 | 0 |
| Pre.hiG | 22 | -16.183 | -8.885 | -2.135 | -10.265 | 8.657 | -27.18 | 0.02 | 0 |
| X1st.hiG | 22 | -9.555 | -3.620 | -1.620 | -5.695 | 5.997 | -22.79 | 0.36 | 0 |
| X2nd.hiG | 20 | -3.790 | -2.050 | -0.580 | -3.356 | 4.401 | -18.35 | 0.10 | 2 |
| X3rd.hiG | 20 | -3.622 | -1.615 | -0.797 | -2.684 | 5.740 | -19.04 | 11.83 | 2 |

Anderson-Darling test for normality

Bartlett test of variances homogeneity p = 1.158681e-06

Normality accepted = * FALSE *

**SIGNIFICANT** difference between groups, p= 3.379486e-05

**Table 38**Wilcoxon rank sum exact test

|  | Pre.lowG | 1st.lowG | 2nd.lowG | 3rd.lowG | Pre.hiG | 1st.hiG | 2nd.hiG |
| --- | --- | --- | --- | --- | --- | --- | --- |
| 1st.lowG | 1.0000000 | NA | NA | NA | NA | NA | NA |
| 2nd.lowG | 0.8113282 | 1.0000000 | NA | NA | NA | NA | NA |
| 3rd.lowG | 0.2791032 | 1.0000000 | 1.0000000 | NA | NA | NA | NA |
| Pre.hiG | 0.0880310 | 0.0807772 | 0.0117821 | 0.0021094 | NA | NA | NA |
| 1st.hiG | 1.0000000 | 0.9677055 | 0.1745549 | 0.0401340 | 1.0000000 | NA | NA |
| 2nd.hiG | 1.0000000 | 1.0000000 | 1.0000000 | 0.1110256 | 0.0868424 | 1 | NA |
| 3rd.hiG | 1.0000000 | 1.0000000 | 1.0000000 | 0.9710741 | 0.0612408 | 1 | 1 |


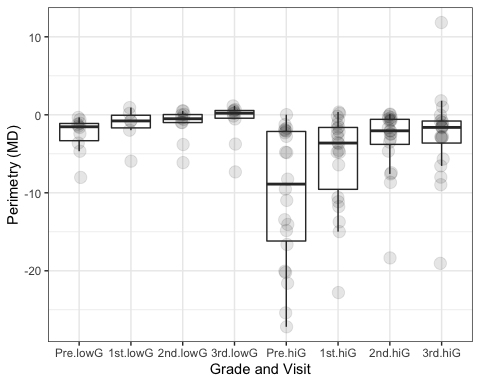


**Figure 32**Box chart plotting perimetry for combinations of the grade and the visit factors. Each dot represents one eye, the box depicts the medina and upper and lower quartile.

# Visual field change

### RNFL

Anderson-Darling test: normality = FALSE

**SIGNIFICANT** correlation between parameters, Spearman rho= -0.4622126 ; p = 0.01327357

*Two tail test*

Effect size and confidence limits, d = -1.04 [ -1.89 -0.19 ]

Power of study with aforementioned effect, pwr = 0.72

MD perim., visit 3-0 [-] vs. RNFL,Visit 0 (um)

**Table 39**Descriptive statistics for the perimetry and RNFL. Parameters listed: N – number of observations, 25q – Lower quartile, Median, 75q – Upper quartile, Mean, SD – Standard deviation, Min – Minimal value, Max – Maximal value, NA – not available.

|  | N | 25q | 50q | 75q | Mean | SD | Min | Max | NA |
| --- | --- | --- | --- | --- | --- | --- | --- | --- | --- |
| MD perim., visit 3-0 [-] | 30 | 0.96 | 2.5 | 9.28 | 6.16 | 7.33 | -0.64 | 25.82 | 2 |
| RNFL,Visit 0 (um) | 30 | 56.25 | 65.0 | 73.00 | 63.93 | 18.11 | 27.00 | 105.00 | 2 |


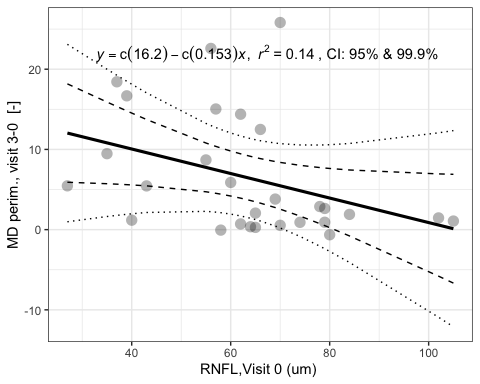


**Figure 33**Scatter plot of the relationship between perimetry outcome after surgery and pre-surgery RNFL. Each dot represents one eye. The linear regression is depicted as a solid line with 95 % (dashed curves) and 99.9 % (dotted curves) confidence intervals.

### GCL

Anderson-Darling test: normality = FALSE

**SIGNIFICANT** correlation between parameters, Spearman rho= -0.6338125 ; p = 0.0002933081

*Two tail test*

Effect size and confidence limits, d = -1.64 [ -2.61 -0.66 ]

Power of study with aforementioned effect, pwr = 0.97

MD perim., visit 3-0 [-] vs. GCL,Visit 0 (um)

**Table 40**Descriptive statistics for the perimetry and GCL. Parameters listed: N – number of observations, 25q – Lower quartile, Median, 75q – Upper quartile, Mean, SD – Standard deviation, Min – Minimal value, Max – Maximal value, NA – not available.

|  | N | 25q | 50q | 75q | Mean | SD | Min | Max | NA |
| --- | --- | --- | --- | --- | --- | --- | --- | --- | --- |
| MD perim., visit 3-0 [-] | 30 | 0.96 | 2.50 | 9.28 | 6.16 | 7.33 | -0.64 | 25.82 | 2 |
| GCL,Visit 0 (um) | 30 | 35.47 | 43.45 | 46.65 | 40.57 | 8.41 | 22.70 | 52.70 | 2 |


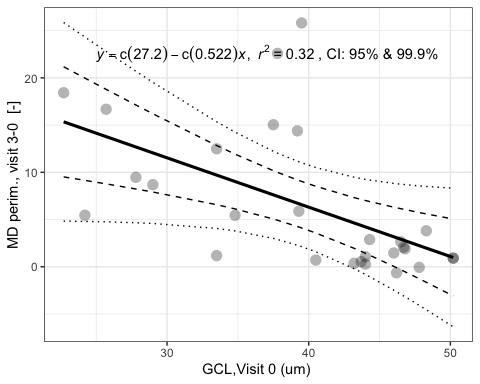


**Figure 34**Scatter plot of the relationship between perimetry outcome after surgery and pre-surgery GCL. Each dot represents one eye. The linear regression is depicted as a solid line with 95 % (dashed curves) and 99.9 % (dotted curves) confidence intervals.

### P100 peak time

Anderson-Darling test: normality = FALSE

**NO** significant relationship, Spearman rho= 0.2780809 ; p = 0.1367721

*Two tail test*

Effect size and confidence limits, d = 0.58 [ -0.18 1.34 ]

Power of study with aforementioned effect, pwr = 0.32

MD perim., visit 3-0 [-] vs. P100 peak time,Visit 0 (ms)

**Table 41**Descriptive statistics for the perimetry and P100 peak time. Parameters listed: N – number of observations, 25q – Lower quartile, Median, 75q – Upper quartile, Mean, SD – Standard deviation, Min – Minimal value, Max – Maximal value, NA – not available.

|  | N | 25q | 50q | 75q | Mean | SD | Min | Max | NA |
| --- | --- | --- | --- | --- | --- | --- | --- | --- | --- |
| MD perim., visit 3-0 [-] | 30 | 0.96 | 2.50 | 9.28 | 6.16 | 7.33 | -0.64 | 25.82 | 2 |
| P100 peak time,Visit 0 (ms) | 32 | 98.18 | 104.55 | 117.98 | 108.89 | 14.89 | 90.00 | 145.20 | 0 |


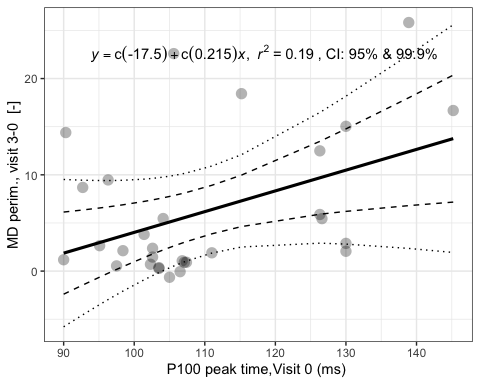


**Figure 35**Scatter plot of the relationship between perimetry outcome after surgery and pre-surgery P100 peak time. Each dot represents one eye. The linear regression is depicted as a solid line with 95 % (dashed curves) and 99.9 % (dotted curves) confidence intervals.

### P100 amplitude

Anderson-Darling test: normality = FALSE

**NO** significant relationship, Spearman rho= -0.2622914 ; p = 0.1610272

*Two tail test*

Effect size and confidence limits, d = -0.54 [ -1.3 0.21 ]

Power of study with aforementioned effect, pwr = 0.29

MD perim., visit 3-0 [-] vs. P100 amplitude,Visit 0 (uV)

**Table 42**Descriptive statistics for the perimetry and P100 interpeak amplitude. Parameters listed: N – number of observations, 25q – Lower quartile, Median, 75q – Upper quartile, Mean, SD – Standard deviation, Min – Minimal value, Max – Maximal value, NA – not available.

|  | N | 25q | 50q | 75q | Mean | SD | Min | Max | NA |
| --- | --- | --- | --- | --- | --- | --- | --- | --- | --- |
| MD perim., visit 3-0 [-] | 30 | 0.96 | 2.50 | 9.28 | 6.16 | 7.33 | -0.64 | 25.82 | 2 |
| P100 amplitude,Visit 0 (uV) | 32 | 1.24 | 1.94 | 3.04 | 2.24 | 1.21 | 0.62 | 5.09 | 0 |


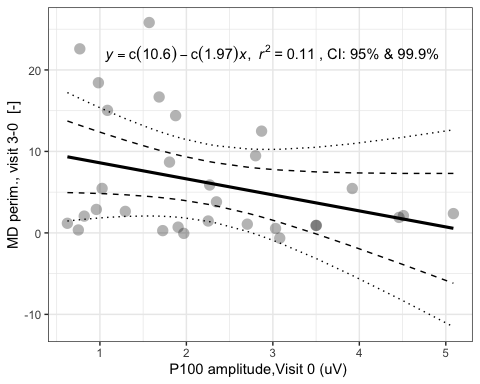


**Figure 36**Scatter plot of the relationship between perimetry outcome after surgery and pre-surgery P100 interpeak amplitude. Each dot represents one eye. The linear regression is depicted as a solid line with 95 % (dashed curves) and 99.9 % (dotted curves) confidence intervals.

### N160 peak time

Anderson-Darling test: normality = FALSE

**NO** significant relationship, Spearman rho= 0.3519912 ; p = 0.06621964

*Two tail test*

Effect size and confidence limits, d = 0.75 [ -0.05 1.56 ]

Power of study with aforementioned effect, pwr = 0.46

MD perim., visit 3-0 [-] vs. N160 peak time,Visit 0 (ms)

**Table 43**Descriptive statistics for the perimetry and N160 peak time. Parameters listed: N – number of observations, 25q – Lower quartile, Median, 75q – Upper quartile, Mean, SD – Standard deviation, Min – Minimal value, Max – Maximal value, NA – not available.

|  | N | 25q | 50q | 75q | Mean | SD | Min | Max | NA |
| --- | --- | --- | --- | --- | --- | --- | --- | --- | --- |
| MD perim., visit 3-0 [-] | 30 | 0.96 | 2.5 | 9.28 | 6.16 | 7.33 | -0.64 | 25.82 | 2 |
| N160 peak time,Visit 0 (ms) | 30 | 156.98 | 172.8 | 184.57 | 172.46 | 19.10 | 137.10 | 218.70 | 2 |


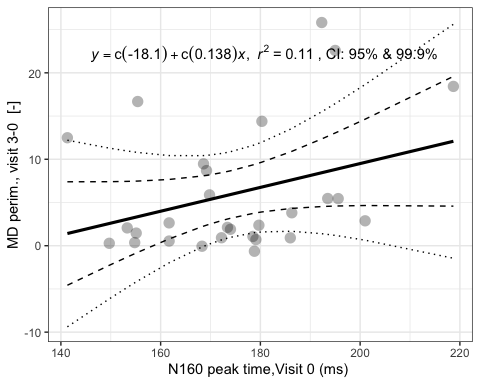


**Figure 37**Scatter plot of the relationship between perimetry outcome after surgery and pre-surgery N160 peak time. Each dot represents one eye. The linear regression is depicted as a solid line with 95 % (dashed curves) and 99.9 % (dotted curves) confidence intervals.

### N160 amplitude

Anderson-Darling test: normality = FALSE

**SIGNIFICANT** correlation between parameters, Spearman rho= -0.3924466 ; p = 0.03974387

*Two tail test*

Effect size and confidence limits, d = -0.85 [ -1.67 -0.03 ]

Power of study with aforementioned effect, pwr = 0.56

MD perim., visit 3-0 [-] vs. N160 amplitude,Visit 0 (uV)

**Table 44**Descriptive statistics for the perimetry and N160 interpeak amplitude. Parameters listed: N – number of observations, 25q – Lower quartile, Median, 75q – Upper quartile, Mean, SD – Standard deviation, Min – Minimal value, Max – Maximal value, NA – not available.

|  | N | 25q | 50q | 75q | Mean | SD | Min | Max | NA |
| --- | --- | --- | --- | --- | --- | --- | --- | --- | --- |
| MD perim., visit 3-0 [-] | 30 | 0.96 | 2.50 | 9.28 | 6.16 | 7.33 | -0.64 | 25.82 | 2 |
| N160 amplitude,Visit 0 (uV) | 30 | 2.70 | 4.01 | 5.05 | 3.86 | 1.48 | 1.18 | 6.95 | 2 |


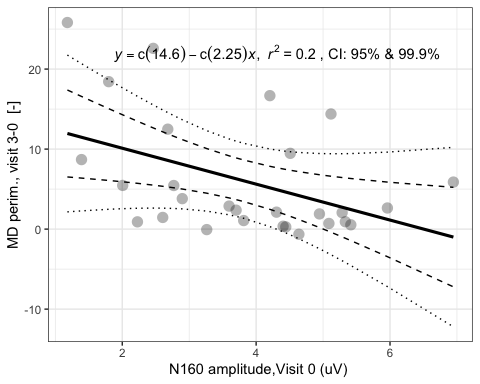


**Figure 38**Scatter plot of the relationship between perimetry outcome after surgery and pre-surgery N160 interpeak amplitude. Each dot represents one eye. The linear regression is depicted as a solid line with 95 % (dashed curves) and 99.9 % (dotted curves) confidence intervals.

### Visual acuity

Anderson-Darling test: normality = FALSE

**SIGNIFICANT** correlation between parameters, Spearman rho= 0.368903 ; p = 0.04484605

*Two tail test*

Effect size and confidence limits, d = 0.79 [ 0.01 1.58 ]

Power of study with aforementioned effect, pwr = 0.53

MD perim., visit 3-0 [-] vs. Visual acuity,Visit 0 (logMAR)

**Table 45**Descriptive statistics for the perimetry and Visual acuity. Parameters listed: N – number of observations, 25q – Lower quartile, Median, 75q – Upper quartile, Mean, SD – Standard deviation, Min – Minimal value, Max – Maximal value, NA – not available.

|  | N | 25q | 50q | 75q | Mean | SD | Min | Max | NA |
| --- | --- | --- | --- | --- | --- | --- | --- | --- | --- |
| MD perim., visit 3-0 [-] | 30 | 0.96 | 2.5 | 9.28 | 6.16 | 7.33 | -0.64 | 25.82 | 2 |
| Visual acuity,Visit 0 (logMAR) | 32 | 0.10 | 0.4 | 0.72 | 0.48 | 0.47 | -0.20 | 1.40 | 0 |


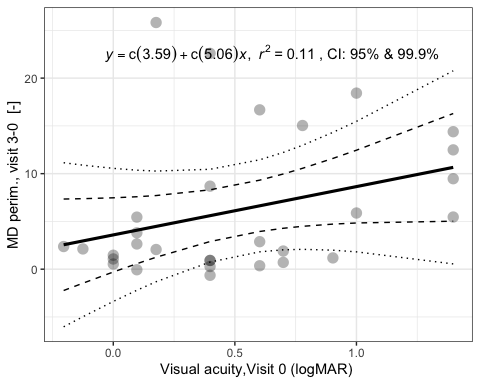


**Figure 39**Scatter plot of the relationship between perimetry outcome after surgery and pre-surgery visual acuity. Each dot represents one eye. The linear regression is depicted as a solid line with 95 % (dashed curves) and 99.9 % (dotted curves) confidence intervals.
